# gespeR: a statistical model for deconvoluting off-target-confounded RNA interference screens

**DOI:** 10.1186/s13059-015-0783-1

**Published:** 2015-10-07

**Authors:** Fabian Schmich, Ewa Szczurek, Saskia Kreibich, Sabrina Dilling, Daniel Andritschke, Alain Casanova, Shyan Huey Low, Simone Eicher, Simone Muntwiler, Mario Emmenlauer, Pauli Rämö, Raquel Conde-Alvarez, Christian von Mering, Wolf-Dietrich Hardt, Christoph Dehio, Niko Beerenwinkel

**Affiliations:** Department of Biosystems Science and Engineering, ETH, Zurich, Switzerland; SIB Swiss Institute of Bioinformatics, Lausanne, Switzerland; Department of Biology, ETH, Zurich, Switzerland; Biozentrum, University of Basel, Basel, Switzerland; Institute for Tropical Health and Departamento de Microbiología y Parasitología, Universidad de Navarra, Pamplona, Spain; Institute of Molecular Life Sciences, University of Zurich, Zurich, Switzerland

**Keywords:** RNAi, siRNA, Off-target, Confounded, Phenotype, Deconvolution, Statistical model, Hit prioritization, Pathogen infection screen

## Abstract

Small interfering RNAs (siRNAs) exhibit strong off-target effects, which confound the gene-level interpretation of RNA interference screens and thus limit their utility for functional genomics studies. Here, we present gespeR, a statistical model for reconstructing individual, gene-specific phenotypes. Using 115,878 siRNAs, single and pooled, from three companies in three pathogen infection screens, we demonstrate that deconvolution of image-based phenotypes substantially improves the reproducibility between independent siRNA sets targeting the same genes. Genes selected and prioritized by gespeR are validated and shown to constitute biologically relevant components of pathogen entry mechanisms and TGF-β signaling. gespeR is available as a Bioconductor R-package.

**Electronic supplementary material:**

The online version of this article (doi:10.1186/s13059-015-0783-1) contains supplementary material, which is available to authorized users.

## Background

The discovery of RNA interference (RNAi) brought the exciting prospect of targeted gene interventions for detailed characterization of biological processes to the functional genomics community. Today, there exist multiple commercial and academic libraries, based on different reagents, such as small interfering RNA (siRNA) or small hairpin RNA (shRNA), for human cell lines and a range of model organisms. However, phenotypic readouts of RNAi knockdown experiments using distinct reagents targeting the same gene exhibit poor reproducibility [1–3]. For siRNAs, this lack of reproducibility is largely due to sequence-dependent off-target effects. The intended on-target gene is silenced through full complementarity of the siRNA to the open reading frame (ORF) of its transcript. Each siRNA, however, silences hundreds of additional off-target genes, which has been shown in vitro [4] and in silico (Additional file 1). Using the microRNA (miRNA) pathway, the set of off-target genes of an siRNA is mainly determined by complementarity of its seed region (positions 2–8) to the 3’ untranslated regions (UTRs) of the transcript [5]. Thus, rather than a single-gene knockdown, an RNAi knockdown experiment is a combinatorial knockdown of multiple genes, where the resulting knockdown phenotype does not directly reveal the effect of individual genes [6, 7].

**Fig. 1:**
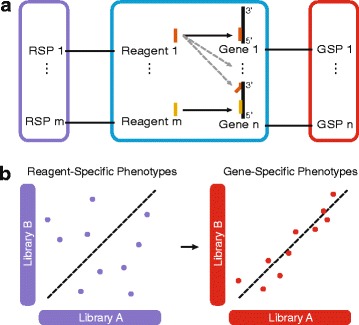
Gene-specific phenotypes (GSPs; *red*) estimated from off-target-confounded RNAi screens. **a** Schematic representation of a knockdown screen. RNAi reagents (e.g., siRNAs) target their intended on-target (*black solid arrow*) and additional off-target (*grey dashed line arrows*) genes. Each gene has a hidden GSP, whereas the observed reagent-specific phenotypes (RSPs; *violet*) correspond to the combined effect of on- and off-target genes. **b** Unlike RSPs, deconvoluted GSPs are expected to exhibit high concordance between distinct libraries containing different reagents targeting the same genes

In fact, despite improved algorithms for the design of RNAi reagents [8], chemical modifications of reagents [9, 10], and the development of computational methods to improve reproducibility and to minimize the risk of reporting false positive hits [11–13], it remains challenging to identify the specific effect of each individual gene on the phenotype from RNAi screens. These limitations dampened initial excitement and raised concerns about the utility of the technology [11, 14]. Here, we address this challenge and introduce gespeR (for *ge*ne-*s*pecific *p*henotype *e*stimato*r*), a statistical model for the estimation of hidden gene-specific phenotypes (GSPs) from observed reagent-specific phenotypes (RSPs). We model the observed RSPs as the weighted sum of individual GSPs from all on- and off-target genes, where the weights are proportional to the strengths of gene knockdowns by a reagent (Fig. 1a). Unlike RSPs, the inferred GSPs are gene-specific, deconvoluted phenotypes, i.e., they are independent of the underlying RNAi library and hence highly reproducible between distinct RNAi libraries (Fig. 1b) and ultimately allow constructing unconfounded gene hit rankings for follow-up analyses.

For the validation of gespeR, we performed extensive in silico and in vitro experiments, including (1) evaluating the performance of our model to predict new, unseen siRNA phenotypes, (2) demonstrating strong reproducibility between GSP estimates from different RNAi libraries, and (3) evaluating the biological significance of estimated GSPs. We applied gespeR to phenotypes derived from three high-content, image-based pathogen infection screens and an additional, previously published screen on transforming growth factor (TGF)-β signaling [6]. Where applicable, the performance of gespeR was compared with established RNAi gene prioritization strategies: in silico pooling (ISP), redundant siRNA analysis (RSA) [15] and haystack [16]. ISP is defined as simple averaging over all RNAi reagent phenotypes for the same gene and subsequent ranking. RSA performs statistical tests for the enrichment of multiple reagents targeting the same gene at the top and bottom of ranked phenotype lists and ranks genes according to *p* values. Haystack uses iterative forward selection of gene transcripts to build a linear model that explains observed phenotypes based on predicted off-target effects. gespeR is related to haystack (see "Comparison of the gespeR and haystack models" section), but is based on elastic net regularization [17] to select and prioritize genes and additionally uses on- and off-targets of RNAi reagents to model observed phenotypes.

## Results and discussion

### The gespeR model for deconvoluting RNAi phenotypes

gespeR can be applied to any RNAi screening data set confounded by off-target effects. The input to the model consists of observed RSPs (e.g., from siRNA, shRNA, or even small-molecule knockdown experiments) and reagent-to-gene target relations. The relationships between each reagent *i* and each gene *j* are summarized in the matrix *X* = (*X*_*ij*_) ∈ [0, 1]^*n* × *p*^. They are typically not provided by library vendors, but can be experimentally determined or, in the case of siRNAs as in this study, predicted using additional tools. We define the *j*-th column of *X*, *X*_*j*_ ∈ [0, 1]^*n*^ as the vector of knockdown strengths of gene *j* for siRNAs *i* = 1, …, *n*. For observed phenotypes 1, …, *k*, we denote by *Y* ∈ ℝ^*n* × *k*^ the matrix of *k* real-valued RSPs for siRNAs *i* = 1, …, *n*. We assume that the conditional expectation of *Y* is linear in *X*_1_, …, *X*_*p*_ and that the deviations of *Y* around its expectation are additive and Gaussian. Hence, the observed RSPs for reagents *i* = 1, …, *n* are modeled as the weighted sum of GSPs *β*_*j*_ ∈ ℝ^*k*^ of all targeted genes *j* = 1, …, *p*:

$$ Y=\mathrm{E}\left(Y\Big|{X}_1,\dots,\;{X}_p\right)+\varepsilon ={\displaystyle \sum_{j=1}^p}{X}_j{\beta}_j+\varepsilon, $$

where the error *ε* ~ Ν(0, *σ*^2^**I**_***k***_). In this study, we exclusively analyze univariate phenotypes (*k* = 1) derived from a single read-out.

### Application to image-based pathogen infection screen phenotypes

We applied gespeR to an extensive data set from high-content, image-based pathogen infection screens designed for the investigation of the entry pathways of three facultative intracellular bacterial pathogens: *Brucella abortus*, *Bartonella henselae*, and *Salmonella typhimurium* (see "Image-based pathogen infection screens" section). In total, we analyzed 115,878 knockdown experiments per pathogen screen using single siRNA and pooled siRNA libraries from Ambion, Dharmacon, and Qiagen (Table 1). The primary phenotype extracted from quantified image features was *Infectivity*, defined as the fraction of infected cells within a well. We removed readouts from outlier wells with low cell count resulting from lethal siRNA transfections, i.e., wells that contained less than 250 cells from approximately 2500–4000 cells expected under normal growth, in order to avoid large variation in the multiplicity of infection (MOI). In addition, we removed outlier wells with less than five infected cells, because we cannot rule out experimental failure in these cases. Row and column plate effects were corrected for using the B score model without applying the smoothing option (Additional file 2) [18]. Phenotypes were scaled per plate using median-absolute deviation (MAD) and aggregated per siRNA by the mean over all replicates.

**Table 1:** siRNA libraries for pathogen infection screens

Vendor	Product	Type	Scope	siRNAs/gene
Ambion	Silencer® Select	Single	Kinome	3
	Silencer® Select	Single	Validation (1,837)	6
Dharmacon	Human ON-TARGETplus	Single	Kinome	4
	Human ON-TARGETplus	Pooled	Genome	4
Qiagen	Human Druggable Genome siRNA V3	Single	Genome	4
	Human Refseq Xm siRNA V1	Single	Predicted mRNA	4
	Human Predicted Genome V1	Single	Predicted mRNA	4

Libraries included kinome-, validation- and genome-wide libraries of different structure (single-siRNA and pooled) and from different vendors

### Model validation and benchmarking

In the absence of a ground truth for cellular signaling of pathogen entry, we validated gespeR in several independent ways and, whenever possible, compared its performance with two established prioritization methods, RSA [15] and haystack [16], as well as with the baseline of RSPs (the observed RSPs) or in silico pooling. We first assessed the predictive power and concordance of phenotypes between distinct libraries, since high predictive power and strong concordance are necessary conditions for meaningful gene-level interpretation of RNAi screens (see "Necessary conditions for model validity" section). Second, we evaluated the biological relevance of prioritized hit genes by inter-pathogen comparisons, gene set enrichment analysis (GSEA), and literature validation of hit lists in two independent biological systems. Note that GSP estimates from convoluted RSPs cannot be directly validated by additional RNAi experiments on the same gene, because it has to be assumed that off-target effects will again confound the validation experiment RSPs.

#### Measures of mutual concordance between ranked lists of phenotypes

In order to capture most important aspects of mutual concordance, in the analyses described below we applied four different measures to compare ranked lists of phenotypes. First, because genes with a strong positive or negative phenotype are of major interest, we determined the rank-biased overlap [19] of the top (rbo_↓_) and bottom (rbo_↑_) of gene lists ranked by their phenotype. In addition, since gespeR, haystack, and RSA, in general, do not necessarily select fully overlapping subsets of relevant genes, we measured the overall relative overlap of selected genes using the Jaccard index (J), which is defined as the cardinality of the intersection divided by the cardinality of the union of two sets. Furthermore, we calculated Spearman’s correlation coefficient (ρ) between phenotypes, which indicates the overall rank similarity. In order to ensure fair comparisons between methods, ranked lists of RSPs and ISPs, respectively, were trimmed to the lengths of the corresponding lists of GSPs estimated by gespeR, keeping the number of selected positive and negative phenotypes fixed.

#### gespeR accurately predicts siRNA knockdown phenotypes

**Fig. 2:**
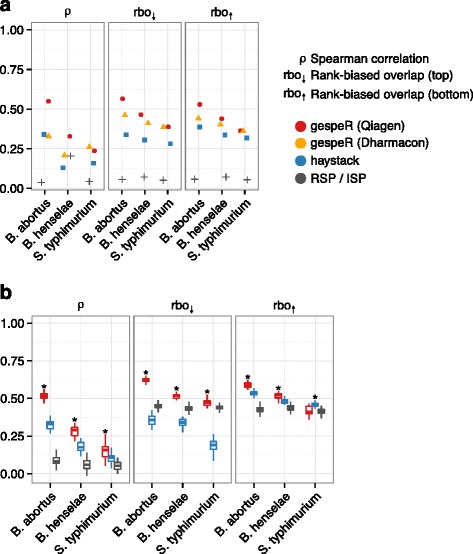
gespeR predicts siRNA phenotypes with significantly higher accuracy than in silico pooling (ISP) and haystack across all pathogens. Mutual concordance is evaluated between predicted and measured reagent-specific phenotypes (RSPs) for the same siRNAs. *Significantly better than second best method (Wilcoxon rank sum test, *p* < 0.05). **a** Phenotypes for 1871 validation screen siRNAs from Ambion were predicted in a blind test prior to experiments and evaluated against eventually measured RSPs. **b** Subsetting seven data points for the kinome-wide data set, RSPs were repeatedly predicted for a training set and evaluated against a disjoint test set

In order to assess the predictive power of our model in a blind test, we predicted combinatorial RSPs for 1871 previously unseen Ambion validation screen siRNAs prior to the validation experiment, and seven kinome-wide data sets. Using gespeR, we first estimated gene-specific phenotypes for all pathogens from the joint set of 90,264 Qiagen siRNAs, denoted GSP_Q,_ and 18,041 Dharmacon siRNA pools, denoted GSP_D_. GSP_Q_s and GSP_D_s were then independently used to compute the expected phenotypes of the unseen screens (Additional file 3). In this analysis, we compared the predictive performance of gespeR only with ISP of RSPs and haystack, because RSA cannot predict RSPs (see “Limited applicability of RSA and haystack” section in Additional file 4). Haystack operates on seed-averaged phenotypes and not on full siRNA phenotypes. Therefore, as an approximation to the prediction of the RSP for a specific siRNA using the haystack model, we predicted matching seed-phenotypes based on haystack’s phenotype estimates from the same Qiagen data set. Performance was evaluated by measuring concordance of predicted against measured RSPs. For the prediction of the new, unseen siRNA phenotypes, gespeR showed higher concordance than predictions from both haystack and ISP as evaluated by correlation between phenotypes and rank-biased overlap of ranked gene lists across all pathogens (Fig. 2a). The predictive performance was stronger for GSP_Q_s for all pathogens, except for *S. typhimurium*, where GSP_D_s performed slightly better, likely due to strain-specific effects from the Qiagen genome-wide screen for *S. typhimurium*.

gespeR also showed significantly better predictive performance than haystack and ISP on kinome-wide phenotypic readout available for seven independent siRNA knockdowns (3× Dharmcon single-siRNA and 4× Dharmacon pooled). We selected from the seven readouts all combinations of two disjoint sets, each of size three. Each time, we held out one set as a test set and used the remaining set for predictions. Using gespeR, combinatorial RSPs were predicted based on GSP_Q_ estimated from Qiagen siRNAs not contained in either of the two sets. For haystack, parameters learned from the same Qiagen data set were used for predictions. ISP predictions were obtained by averaging over all phenotypes from reagents targeting the same gene. Predictions were compared with the mean phenotype of the test set and performance was evaluated using the measures of concordance described above (Fig. 2b).

#### gespeR GSPs are highly concordant between distinct libraries

**Fig. 3:**
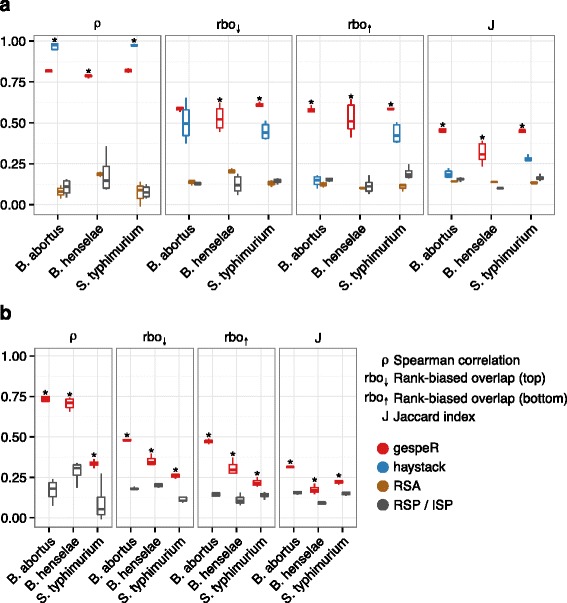
Gene-specific phenotypes (GSPs) estimated by gespeR are highly reproducible between different RNAi libraries across all pathogens. Mutual concordance is evaluated between phenotypes for the same genes. *Significantly better than second best method (Wilcoxon rank sum test, *p* < 0.05). **a** gespeR GSPs for four Qiagen genome-wide sub-libraries are significantly more reproducible than RSPs and estimates from haystack and RSA. **b** gespeR GSPs exhibit significantly stronger concordance than in silico pooled RSPs (ISPs) between single and pooled siRNA libraries from different vendors (Qiagen single-siRNA versus Dharmacon pooled)

We investigated the concordance between phenotypes from different sets of siRNAs targeting the same genes. The set of Qiagen siRNAs was split into four distinct genome-wide sub-libraries, each containing one siRNA per gene, and GSPs were separately estimated for each sub-library (GSP_Q,1_, …, GSP_Q,4_). For RSA, we created all possible combinations of two libraries and compared (1,2) versus (3,4), (1,3) versus (2,4), and (1,4) versus (2,3) (see “Limited applicability of RSA and haystack” section in Additional file 4). Concordance of GSPs between all pairs of sub-libraries was evaluated as described above (see "Measures of mutual concordance between ranked lists of phenotypes" section) and compared with haystack, RSA, and RSPs (Fig. 3a). We found RSPs to exhibit low correlation (median ρ = 0.14) and rank-biased overlap (median rbo_↓_ = 0.16, rbo_↑_ = 0.18), confirming that even sets of strongly negative or positive observed phenotypes do not agree between different siRNAs targeting the same gene. Experimental noise alone cannot account for this poor reproducibility, as correlation between technical replicates (repeated knockdowns using the same siRNA) was much higher (0.8 on average) [20]. Mutual concordance between estimated GSPs obtained from gespeR were close to the level of concordance between technical replicates and, compared with RSPs, up to five times higher with respect to correlation and rbo for both the top and bottom of ranked gene lists. Corresponding pairwise distributions of GSPs and RSPs are provided in Additional files 5 and 6. Haystack estimates showed strikingly high correlation between sets of selected genes, although at the cost of extremely small sets of mutually selected genes between different sub-libraries (median J = 0.25). The median number of mutually selected genes for haystack was m = 13 genes, while we could not evaluate concordance for *B. henselae* due to a median overlap of m = 2. In contrast, the overall relative overlap between sets of genes selected by gespeR is significantly higher (J = 0.44, m = 1093.5 genes), indicating a more stable and comprehensive gene selection procedure. Moreover, gespeR showed significantly higher rbo than haystack, for both the top and the bottom of ranked gene lists. gespeR also outperforms RSA across all measures of concordance. RSA did not show significant improvement over RSPs, which may be due to the fact that RSA strongly depends on the number of reagents per gene.

GSPs estimated by gespeR also showed significantly higher overall concordance between different library types (all genome-wide, pooled versus single siRNA) and vendors (Qiagen versus Dharmacon) compared with RSPs (Fig. 3b). Performance was worse for *S. typhimurium*, likely due to the use of different HeLa cell lines and slightly different pathogen strains in the Qiagen and Dharmacon screens (Table 1). We omitted comparisons to haystack and RSA, as both methods are not applicable to the pooled siRNA library (see “Limited applicability of RSA and haystack” section in Additional file 4). In order to rule out model biases as the underlying source of reproducibility, we showed that gespeR GSPs estimated from randomized RSPs and from randomized siRNA-to-gene target relations did not show concordance (see “Application of gespeR to randomized data” section in Additional file 4 and Figure S6a in Additional file 7). We also confirmed that GSPs estimated by gespeR are not biased towards specific transcript features such as GC content or the number of nucleotides (Figure S6b in Additional file 7).

#### Single-siRNA libraries yield better model fits

Model fits for Qiagen sub-libraries, and the pooled Dharmacon library were appreciable for *B. abortus* and *S. typhimurium*, with a coefficient of determination (R^2^) of over 0.5 for the Qiagen libraries and over 0.35 for the Dharmacon library. The fits for *B. henselae* showed slightly lower R^2^ values of around 0.25 for both library types (Additional file 8). This indicates that, given the same number of siRNAs, gespeR yields higher performance on phenotypic data stemming from single-siRNA libraries than pooled libraries.

#### Inter-pathogen comparison of GSPs results in the biologically expected pattern

**Fig. 4:**
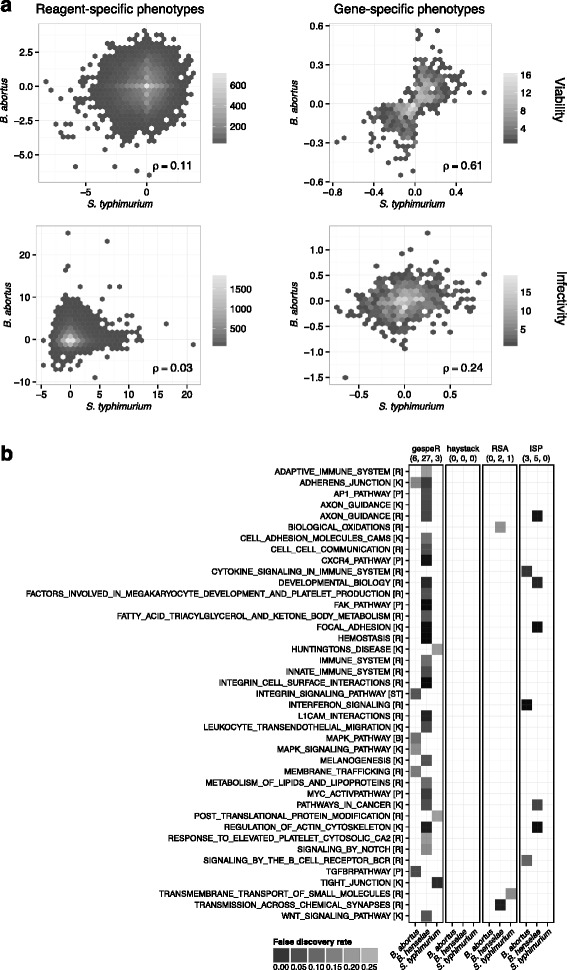
Gene-specific phenotypes (GSPs) for pathogen entry estimated by gespeR from two distinct genome-wide Qiagen sub-libraries are biologically meaningful. **a** Scatterplots of reagent-specific and estimated gene-specific phenotypes between the pathogens *B. abortus* and *S. typhimurium* for *Infectivity* and the auxiliary phenotype of *Viability*. Unlike RSPs, GSPs exhibit biologically expected high correlation between (pathogen-independent) *Viability* phenotypes and only low to moderate correlation for *Infectivity*. **b** Gene set enrichment analysis: pathways significantly enriched at a false discovery rate (FDR) smaller than 0.25 for decreased *Infectivity* and gene lists from gespeR GSPs, haystack, RSA, and ISPs for all pathogens. Canonical pathway databases: *R* Reactome, *K* KEGG, *ST* Signal transduction KE. Pathways, such as focal adhesion or integrin- and TGF-β-signaling, shown to play a crucial role in pathogen entry, are enriched exclusively for GSPs; 62.5 % of pathways enriched for ISPs are also enriched for GSPs. RSA gene rankings are exclusively enriched for three pathways, while haystack rankings did not show sufficient overlap with any tested gene set (minimum overlap n = 15)

In order to assess inter-pathogen concordance between GSPs estimated by gespeR, we compared GSP_Q_ estimates for *B. abortus* and *S. typhimurium* for *Infectivity* and the auxiliary phenotype of *Viability*, defined as the normalized cell count after infection, between two distinct genome-wide Qiagen sub-libraries (Fig. 4a). Unlike RSPs, GSPs showed strong correlation for *Viability* and weak correlation for *Infectivity*. This phenomenon is biologically expected, because gene knockdowns with a strong effect on the growth rate of cells (*Viability*) are largely pathogen-independent. *Infectivity*, in contrast, is pathogen dependent and subtle correlation may be explained by a few shared components between different pathogen entry mechanisms.

#### GSEA reveals biologically relevant pathways for pathogen entry

GSEA was performed in order to test for enrichment of known biological pathways based on ranked gene lists from gespeR, haystack, RSA, and ISP [21]. We deployed the *GSEAPreranked* module from the Broad Institute GSEA suite [22] and used (estimated) phenotypes as the ranking statistic with default parameters and 1000 permutations. Enrichment was tested against the Canonical Pathway database (c2.cp.v4.0.entrez.gmt) downloaded from MSigDB [21]. GSEA results are visualized using a heat map representation of significantly enriched pathways (Y-axis) for each pathogen (X-axis) grouped in panels for each method (Fig. 4b). All pathways enriched for at least one method-pathogen combination with a false discovery rate smaller than 0.25 are shown. We focused on decreased *Infectivity* phenotypes, the phenotype of primary interest for follow-up studies indicating a repressive role in pathogen infection. gespeR identified 37 significantly enriched pathways in total for all studied pathogens, including a number of pathways exclusively enriched for GSPs, which were previously reported to play a role in infection [23, 24], such as focal adhesion, integrin-signaling, and TGF-β signaling. Five out of eight (62.5 %) pathways enriched for ISPs are also enriched for gespeR GSPs. For RSA, three pathways are enriched, not overlapping with any other method, while for haystack, the threshold of a default minimum overlap of n = 15 genes was not met for any tested gene set, resulting in no significant enrichment.

#### Top-ranked GSPs are enriched for canonical hits for pathogen entry

Investigation of the top 50 hit genes ranked according to the absolute gene-specific phenotype provides additional evidence for the biological relevance of GSPs estimated by gespeR for the three investigated pathogens (Tables S1–S3 in Additional file 4). The hit lists contain several genes of previously reported pathways, for instance, genes related to actin dynamics and the COPI complex for *B. abortus* (*CDC42*, *ARF1*), integrin signaling and invasome formation for *B. henselae* (*ITGA5*, *ITGB1*), as well as components related to the *S. typhimurium* entry mechanism (*MYH9*, *IQGAP1*). The distribution of *B. henselae* GSPs exhibits lower variation compared with GSP estimates for both other pathogens. This observation can be attributed to the fact that RSP input to our model for *B. henselae* also showed lower variation (Additional file 9).

#### gespeR identifies hits for TGF-β signaling in independent RNAi data set

We validated gespeR on another, previously published RNAi screen designed to detect members of the TGF-β pathway in a human keratinocyte cell line using a GFP-SMAD2 reporter fusion protein [6]. This screen had been reported to suffer severely from confounding off-target effects. gespeR identified the two main upstream modulators *TGFBR1* and *TGFBR2*, together with additional components of the pathway, such as *SMURF1*, as well as their respective roles as positive and negative regulators among the top ten genes (Table S4 in Additional file 4). While the two upstream modulators were successfully identified in the original study, *SMURF1* as a negative regulator was not. When tested against the KEGG (Kyoto Encyclopedia of Genes and Genomes) pathway for TGF-β signaling (hsa04350), gespeR yielded larger overlap with its top-100 predicted hits (eight genes) than both haystack (one gene) and RSA (four genes). In addition to TGF-β signaling, GSEA revealed that gespeR’s ranked GSP estimates are significantly enriched for four further pathways, while no enrichment was found for haystack, RSA, or ISP (Additional file 10).

## Conclusions

Despite substantial evidence of sequence-dependent, miRNA-like off-target silencing in RNAi screens [14], the problem has been widely ignored in the field of functional genomics for a long time. This shortfall has resulted in high numbers of reported false positive hits and virtually no overlap between similar siRNA-based intervention studies [25]. Recently, a shift in the field led to the development of off-target-aware computational methods to analyze RNAi screens [16, 26, 27]. With gespeR, we provide a statistical framework to correct for confounding off-target effects and to infer deconvoluted, GSPs. We have shown on different RNAi data sets from image-based pathogen infection screens and a reporter protein-based TGF-β-signaling screen that inferred GSPs are, unlike observable RSPs, highly reproducible between different siRNA libraries. In order to test for the reproducibility between ranked lists of phenotypes, we compiled a set of measures that capture different aspects of concordance between ranked lists of genes. gespeR was benchmarked against three established methods commonly used for the analysis of RNAi phenotypes (RSA, haystack, and ISP) and shown to yield superior performance.

In a typical siRNA screening study, a major challenge is the evaluation of biological relevance for prioritized genes, because off-target effects again confound validation experiments that use additional siRNAs. We have avoided this pitfall and were able to predict the outcomes of new phenotypes from validation experiments before they were performed. In general, we (1) show that gespeR successfully predicts combinatorial RSPs based on previously estimated GSPs and target relations for novel siRNAs and (2) provide evidence that prioritized genes are biologically meaningful when performing inter-pathogen comparisons, enrichment analyses, and manual literature validation for top ranked genes. In the future, CRISPR/Cas9 nuclease-based genome editing [28] may become a suitable tool to experimentally validate inferred GSPs but, to date, the technology is not established as a high-throughput technique.

Our study demonstrates that gespeR is a useful tool for the analysis of RNAi data sets. While in the present study we have focused on analyzing data harboring univariate phenotypes, the statistical model and implemented inference algorithm can also be applied to higher-dimensional phenotypes, e.g., those derived from high-content image-based screens. Allowing for unbiased gene-level interpretation of RNAi screens, gespeR significantly improves the selection and prioritization of genes for follow-up analyses and advanced downstream models, e.g., for perturbation-based network reconstruction [29, 30].

## Materials and methods

### Reagent-to-gene target relations *x*_*ij*_

Several current miRNA target prediction tools [31–33] can be employed to predict miRNA-like sequence-dependent off-targets for siRNAs. In this study, we used TargetScan [31] version 6.2 to predict siRNA-to-gene target relations. TargetScan is a linear regression model on gene expression fold change from miRNA sequence features. Its so-called context + scoring [34] considers seed-pairing stability and high target site abundance, which allows for quantitative prediction of gene transcript suppression due to siRNA off-targeting with appreciable performance (see supplementary material of [34]). For our application, we removed the TargetScan feature of using conservation between miRNAs, because it is not applicable to exogenous RNAi reagents. We predicted induced fold changes of expression based on 3’ UTR sequences obtained from the HeLa genome [35]. Let *f*_*ij*_ be the predicted log_2_ fold-change of gene *j* upon transfection with siRNA *i*. Then we define the strength of knockdown by siRNA *i* of gene *j* as ${X}_{ij}=1-{2}^{f_{ij}}$ with 0 ≤ *X*_*ij*_ ≤ 1. In this way, large *X*_*ij*_ values correspond to strong inhibition of target *j* by siRNA *i*. On average, TargetScan predicts around 2000 off-targets per siRNA, including many off-target relations that result only in minor changes of transcript abundance (Additional file 1).

Full 19-nucleotide complementarity matches of an siRNA to a transcript were identified using BLAST and were set to *X*_*ij*_ = 0.75, following a manufacturer’s statement of at least 75 % reduction of expression of intended on-targets [36]. The exact choice of this value did not affect model performance appreciably (Additional file 11), most likely because of dominating off-target effects (Additional file 12) [6, 37].

Transcript level scores predicted by TargetScan were aggregated to gene level by the arithmetic mean over all scores from transcripts corresponding to the same gene. For pooled libraries, we predicted off-target relations for each individual siRNA in the pool and computed the joint off-target relations of the pool as the maximum of all individual siRNAs in the pool. Using the maximum effect of all individual siRNAs in the pool, rather than the arithmetic mean of effects, led to an overall increase in concordance (Additional file 13).

We showed that our method is stable with respect to numerous alterations of the siRNA-to-gene target relation matrix, which disturb the original TargetScan predictions. For instance, we evaluated the performance of our model after adding increasing amounts of noise, or false positive and false negative target relations to the matrix, binarizing the matrix, changing the strength of the on-target effect, or keeping only strong off-target predictions above a certain threshold (see “gespeR’s performance under alterations to the siRNA-to-gene target relation” section in Additional file 4 and Additional file 11).

### Model inference

The linear regression model is fit using elastic net regularization [17] with a group-lasso penalty [38], such that:

$$ \widehat{\beta}=\underset{\beta \in {\mathrm{\mathbb{R}}}^{p\times k}}{\mathrm{argmin}}\frac{1}{2n}{\displaystyle \sum_{i=1}^n}{\left\Vert {y}_i-{\beta}^T{x}_i\right\Vert}_F^2+\lambda \left[\left(1-\alpha \right){\left\Vert \beta \right\Vert}_F^2/2+\alpha {\displaystyle \sum_{j=1}^p}{\left\Vert {\beta}_j\right\Vert}_2\right] $$

where *y*_*i*_ is the *i*-th row of the *n* × *k* response matrix and *β*_*j*_ is the *j*-th row of the *p* × *k* coefficient matrix *β*. We place a group-lasso penalty on each coefficient *k*-vector *β*_*j*_ for a single predictor *x*_*j*_. For *k* > 1 the group-lasso penalty enforces all coefficients for a predictor to be zero or nonzero together, whereas for *k* = 1 (univariate response), the penalty degenerates to the normal lasso. The parameter λ determines the amount of regularization and *α* is the mixing parameter between the ridge and lasso penalty with 0 ≤ *α* ≤ 1. The elastic net penalty selects variables (genes) like the lasso, and shrinks together the coefficients of correlated predictors like ridge. This allows for a sparse solution of GSPs, while retaining simultaneous selection of genes with similar RNAi reagent binding patterns in their respective 3’ UTRs. In addition to this biological motivation for using elastic net regularization, small-scale screens, with only a few hundred RSPs for thousands of genes, lead to underdetermined systems, unidentifiable without regularization. We performed tenfold cross-validation to estimate the parameter λ using the mean-squared error (MSE) as loss function and fixed α at 0.5.

### Necessary conditions for model validity

Concordance between estimated phenotypes for the same gene and the ability of the model to predict unseen phenotypes are necessary conditions for not rejecting the model prior to any follow-up analysis. Indeed, first, if phenotypes estimated from two independent sets of RNAi reagents targeting the same gene are not concordant, it is impossible to unambiguously identify the impact of the respective gene on the phenotype. Hence, only concordant phenotypes can yield reliable results that are interpretable on the single-gene level. Second, the fact that a model fails to predict unseen data indicates that it was misspecified or poorly fitted to the training dataset, and that it does not accurately capture the structure inherent to the modeled system.

### Comparison of the gespeR and haystack models

Haystack is related to gespeR in the sense that it is also a regression model with similar assumptions of combinatorial siRNA phenotypes. The major improvement of gespeR over haystack is a refined variable (i.e., gene) selection procedure. Forward variable subset selection, as applied in haystack, is a discrete and greedy process and therefore exhibits high variance, which is reduced in our model by using elastic net shrinkage [17]. The haystack method models observed combinatorial phenotypes based on off-target effects alone. In contrast, gespeR models combinatorial RSPs based on both on- and off-target effects. In addition, gespeR uses a more involved methodology for siRNA off-target prediction, relying on the TargetScan context + score, considering additional features, such as seed-pairing stability and target abundance in addition to the basic linear model [34]. Moreover, gespeR does not average over phenotypes stemming from siRNAs with the same seed prior to model fitting, maintaining information from individual siRNAs. In contrast to haystack, gespeR can be applied to multivariate phenotypes, e.g., stemming from image-based screens.

### Image-based pathogen infection screens

High-content, image-based pathogen infection screens were performed in the following steps: (1) siRNA reverse transfection, (2) pathogen-specific infection, (3) cell fixation and staining, and (4) microscopic imaging and feature extraction, as described in [20]. For each pathogen, the data are composed of knockdown phenotypes from a genome-wide pooled library from Dharmacon, its kinome-wide single-siRNA counterpart (each with four siRNAs per gene), a genome-wide single-siRNA library from Qiagen (four siRNAs per gene), and 1871 additional single-siRNA validation screen siRNAs from Ambion (Table 1). All screens were performed in HeLa ATCC CCL-2 cells, except for the genome-wide Qiagen screens for *S. typhimurium* (HeLa cell clone Kyoto cells). In this study, we focus on the infectivity phenotype, i.e., the fraction of infected cells per well. This phenotype is constructed from image features, described in detail in [20].

### gespeR Bioconductor package

The gespeR model, including functionality for efficient, parallelized fitting of large data sets, phenotype concordance evaluation, stability selection, and visualization, is available from Bioconductor as the package *gespeR* [39]. The package is released under the GNU General Public License (GPL) version 3 and contains a vignette with an exemplary data analysis workflow, as well as a simulated example data set.

### Availability of supporting data and software

siRNAs and phenotypic readout from the pathogen infection screens are available through PubChem [PubChem:1117357]. Pre-computed siRNA-to-gene target relation matrices for Ambion, Dharmacon, and Qiagen libraries used in this study (Table 1) and for the library from Schultz and co-workers [6] are available online at [40]. In addition, scripts facilitating the automation of TargetScan predictions for the generation of siRNA-to-gene target relations for large collections of siRNAs, including step-by-step instructions, are available from the same website.

## Additional files

Additional file 1: Figure S1. Distribution of the number of off-targets per siRNA for different strength cutoffs on the *Xij* reagent-to-target values. Data are shown for the joint set of all four Qiagen unpooled sub-libraries. On average, each siRNA is predicted to bind around 2000 sequence-dependent off-targets. (PDF 7 kb)

Additional file 2: Figure S2. Removal of row and column effects using B score normalization [18] illustrated for a 384-well plate from the *B. abortus* screen. Columns 1, 2, 23, and 24 contain controls and were removed. B score normalized plates (*bottom*) do not exhibit strong column and row effects, as seen in the observed data (*top*), e.g., in row P or column 15. (PDF 207 kb)

Additional file 3: Figure S3. Prediction of RSPs using the gespeR model. Reagent-specific phenotypes Y^(p)^_i_ are predicted by matrix multiplication of GSP estimates β^(m)^_j_ from one data set (*top*) with reagent-specific target relations X^(p)^_ij_ for another data set (*bottom*). (PDF 298 kb)

Additional file 4: **Supplementary material [** 15 **,** 16 **,** 31 **,** 34 **,** 41 **,** 42 **].** **Table S1** Top 50 hits for *Brucella abortus* ranked by absolute value of *Infectivity* GSPs. PubMed PMIDs are provided for previously reported components. **Table S2** Top 50 hits for *Bartonella henselae* ranked by absolute value of *Infectivity* GSPs. PubMed PMIDs are provided for previously reported components. **Table S3** Top 50 hits for *Salmonella typhimurium* ranked by absolute value of *Infectivity* GSPs. PubMed PMIDs are provided for previously reported components. **Table S4** Top 50 hits for regulators of TGF-β signaling ranked by absolute value of GSPs. PubMed PMIDs are provided for previously reported components. gespeR identifies known components of the TGF-β pathway not identified in the original study due to confounding off-target effects. (DOCX 49 kb)

Additional file 5: Figure S4. Pairwise GSP comparisons reveal high concordance between four Qiagen sub-libraries. Lighter color indicates higher number of points per hexagon. (PDF 429 kb)

Additional file 6: Figure S5. Pairwise RSP comparisons reveal low concordance between four Qiagen sub-libraries. Lighter color indicates higher number of points per hexagon. (PDF 474 kb)

Additional file 7: Figure S6. gespeR GSPs are not concordant for randomized data and do not correlate with GC content or length of 3’ UTR transcripts. a Concordance between gene-specific phenotypes estimated from randomized siRNA-to-gene target relation matrices (covariate matrices; *left*), and from observed siRNA-specific phenotypes (response vectors; *right*). Correlation and rank-biased overlap were throughout close to zero, indicating that no spurious concordance is introduced when gespeR is fit. Results shown are from the *B. abortus* Qiagen screen, but were similar for other pathogens and libraries. b Correlation between estimated gene-specific phenotypes (GSPs) and GC content (*left*) and length of transcript 3' UTRs (*right*). Across libraries, estimated phenotype correlation was close to zero, indicating that GC content and the length of 3' UTRs do not confound the estimation of GSPs. (PDF 145 kb)

Additional file 8: Figure S7. Coefficients of determination (R^2^) for gespeR GSP estimates from data from Qiagen unpooled libraries and the Dharmacon pooled library indicate respectable model fits. (PDF 5 kb)

Additional file 9: Figure S8. Distributions for both GSPs and RSPs exhibit smaller variation for *B. henselae* compared with *B. abortus* and *S. typhimurium*. (PDF 251 kb)

Additional file 10: Figure S9. Gene set enrichment analysis of GSP estimates for regulators of TGF-β signaling reveals five significantly enriched pathways with a false discovery rate (FDR) smaller than 0.25. Canonical pathway databases: *R* Reactome, *K* KEGG. GSPs estimated by gespeR are enriched for five pathways, including, as expected, TGF-β signaling. (PDF 127 kb)

Additional file 11: Figure S10. Various alterations to gespeR’s reagent-to-target relation matrix reveal stability with respect to concordance between GSP estimates from different libraries. The tested variants of the reagent-to-target relation matrix, all of which disturb the original TargetScan predictions, include: *QU_SEEDMATCH*, binary seed-to-3’ UTR match indicator matrix which contains a 1 if the seed of an siRNA matches a 3’ UTR and 0 otherwise; *QU_BINARY*, binarized baseline Qiagen matrices; *QU_TH0x*, thresholded matrices, with only off-targets stronger than 0.x included; *QU_D0*, on-target component removed; *QU_D1*, on-target component set to 100 % knockdown efficacy; *QU_D3o4*, randomly kept three or four out of four on-target components at 75 % and set the remaining ones to 0; *QU_Drnorm*, sampled on-target components from N(0.75, 0.1); *QU_FPFN0x*, swapped 10× percentage of predicted targets with predicted non-targets; *QU_N0x005*, added N(0.x, 0.05) Gaussian noise added to all predicted targets. (PDF 65 kb)

Additional file 12: Figure S11. Off-targeted genes dominate observed reagent-specific phenotypes. The gespeR model was fit to the *B. abortus* phenotypes from the Qiagen screen. On- and off-target contributions to the observed RSP for each reagent i = 1 … n were calculated as c_on,i_ = x_ij_ * β for the on-targeted gene j and c_off,i_ = Y_i_ – c_on,i_ – ε_i_. The distribution of c_off,i_ – c_on,i_ exhibits a strong positive tail, indicating that, in general, the combined contribution to the RSP from all off-targeted genes exceeds the contribution from the on-targeted gene. (PDF 98 kb)

Additional file 13: Figure S12. Maximum aggregation of joint off-target effects for siRNA pools leads to increased concordance compared with arithmetic mean aggregation. (PDF 146 kb)

## Abbreviations

gespeR: gene-specific phenotype estimator
GSEA: gene set enrichment analysis
GSP: gene-specific phenotype
ISP: in silico pooling
KEGG: Kyoto Encyclopedia of Genes and Genomes
miRNA: microRNA
ORF: open reading frame
rbo: rank-biased overlap
RNAi: RNA interference
RSA: redundant siRNA analysis
RSP: reagent-specific phenotype
shRNA: small hairpin RNA
siRNA: small interfering RNA
TGF: transforming growth factor
UTR: untranslated region
