# Erratum to: gespeR: a statistical model for deconvoluting off-target-confounded RNA interference screens

**DOI:** 10.1186/s13059-015-0807-x

**Published:** 2015-10-21

**Authors:** Fabian Schmich, Ewa Szczurek, Saskia Kreibich, Sabrina Dilling, Daniel Andritschke, Alain Casanova, Shyan Huey Low, Simone Eicher, Simone Muntwiler, Mario Emmenlauer, Pauli Rämo, Raquel Conde-Alvarez, Christian von Mering, Wolf-Dietrich Hardt, Christoph Dehio, Niko Beerenwinkel

**Affiliations:** Department of Biosystems Science and Engineering, ETH, Zurich, Switzerland; SIB Swiss Institute of Bioinformatics, Basel, Switzerland; Department of Biology, ETH, Zurich, Switzerland; Biozentrum, University of Basel, Basel, Switzerland; Institute for Tropical Health and Departamento de Microbiología y Parasitología, Universidad de Navarra, Pamplona, Spain; Institute of Molecular Life Sciences, University of Zurich, Zurich, Switzerland

After the publication of this work [1], some content was missing from the Acknowledgements section. The corrected Acknowledgements section is given below:
