# Supplementary figures and images for: gespeR: a statistical model for deconvoluting off-target-confounded RNA interference screens
Source: Genome Biol. 2015 Oct 7;16:220. doi: 10.1186/s13059-015-0783-1 (PMC4597449; doi:10.1186/s13059-015-0783-1)

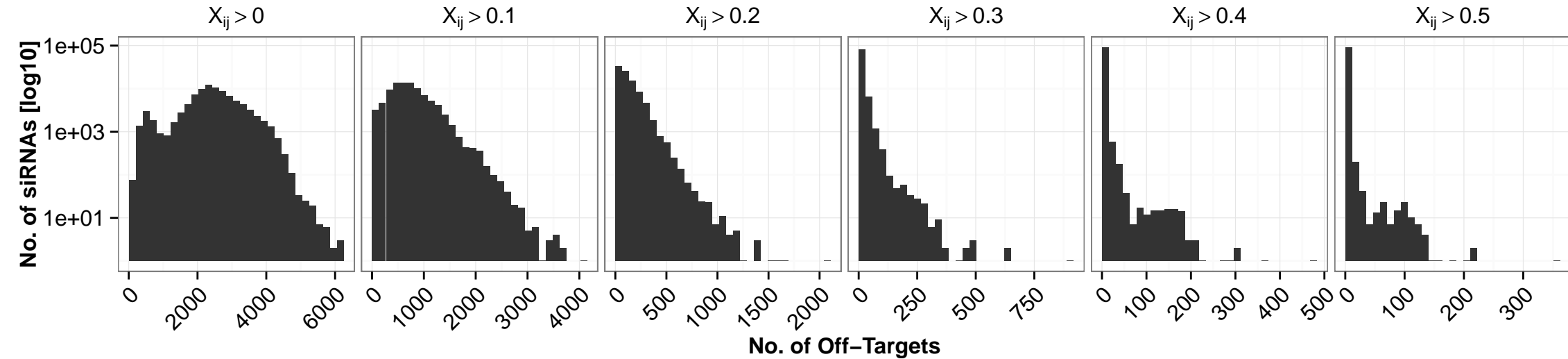

Supplement: Supplementary file 1 — Distribution of the number of off-targets per siRNA for different strength cutoffs on the Xij reagent-to-target values. Data are shown for the joint set of all four Qiagen unpooled sub-libraries. On average, each siRNA is predicted to bind around 2000 sequence-dependent off-targets. (PDF 7 kb) [file 13059_2015_783_MOESM1_ESM.pdf]

Raw Measurements

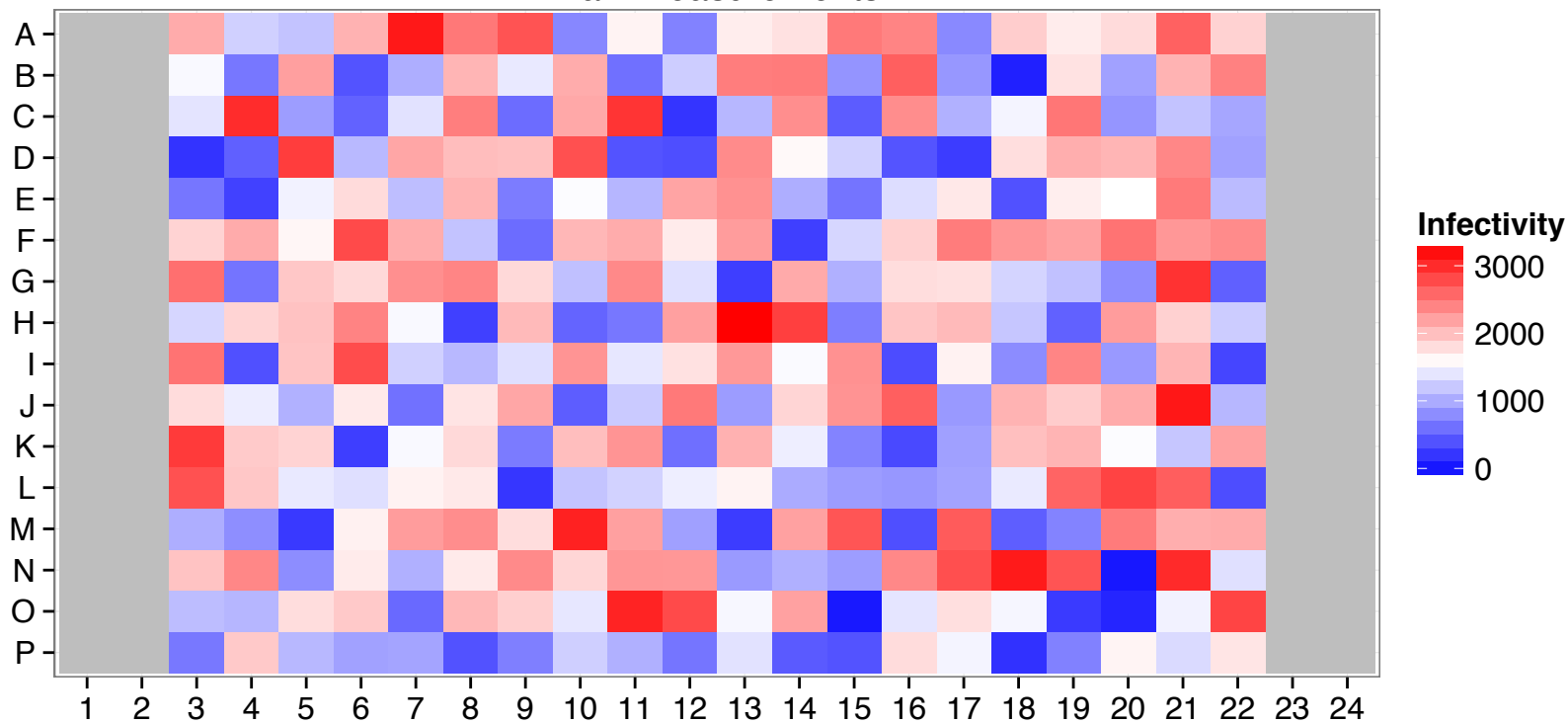

B-Score Normalization

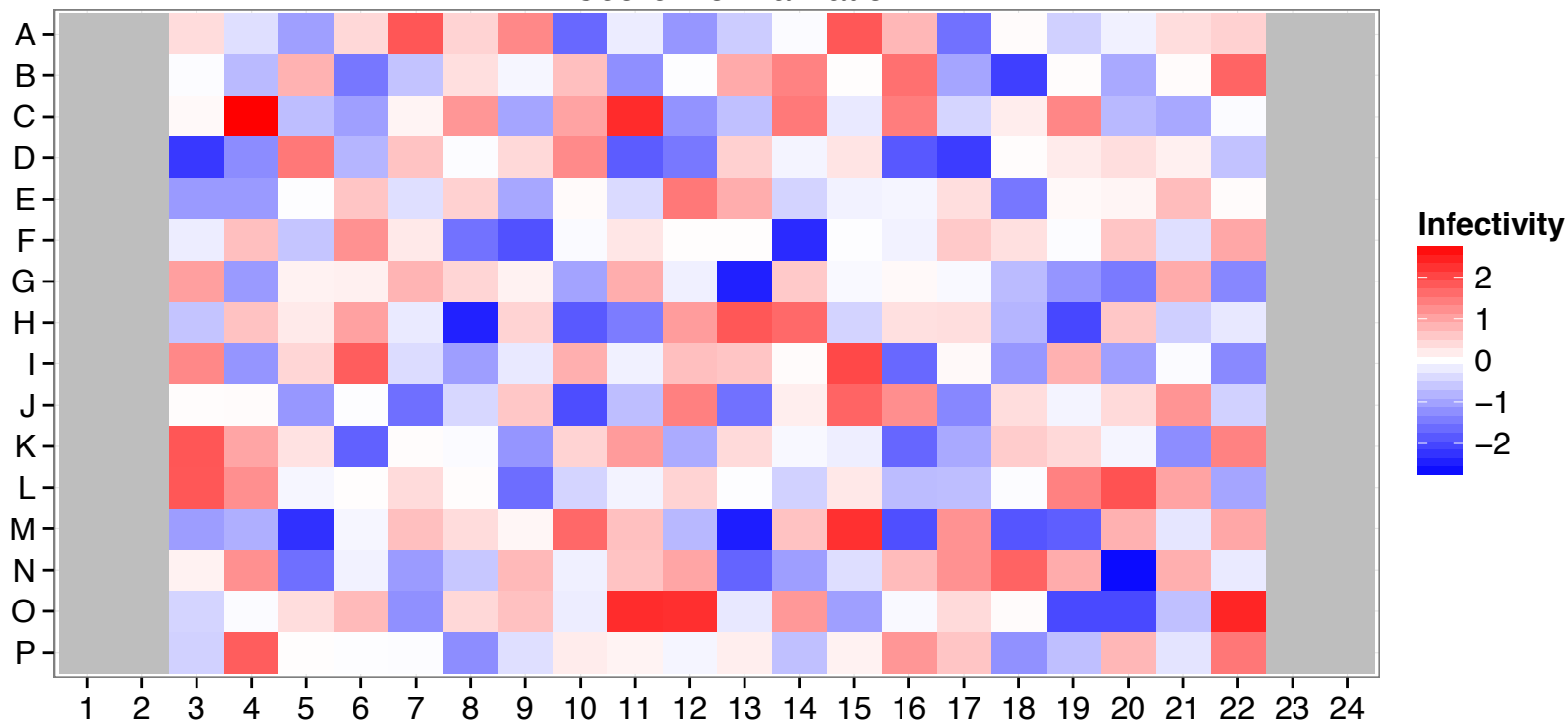

Supplement: Supplementary file 2 — Removal of row and column effects using B score normalization [18] illustrated for a 384-well plate from the B. abortus screen. Columns 1, 2, 23, and 24 contain controls and were removed. B score normalized plates (bottom) do not exhibit strong column and row effects, as seen in the observed data (top), e.g., in row P or column 15. (PDF 207 kb) [file 13059_2015_783_MOESM2_ESM.pdf]

## B. abortus

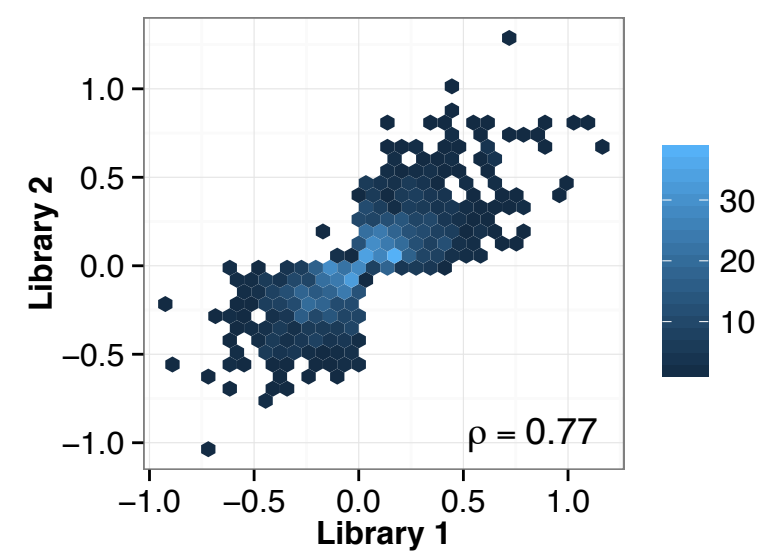

B. henselae

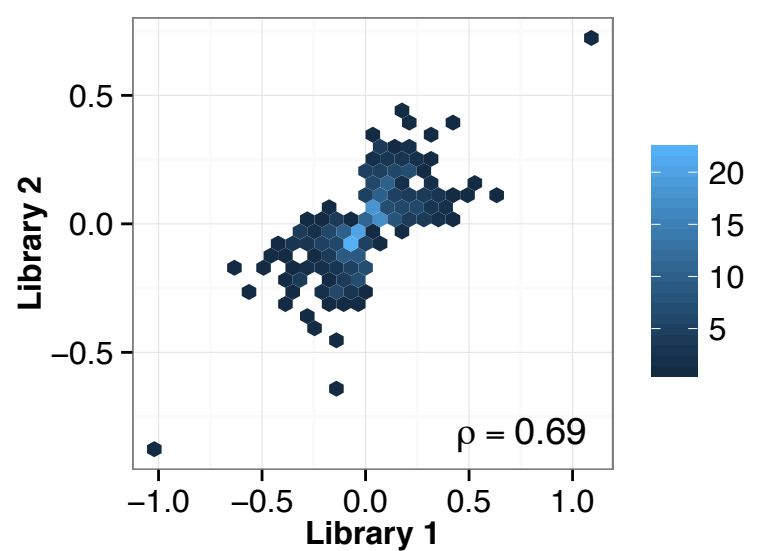

*S. typhimurium*

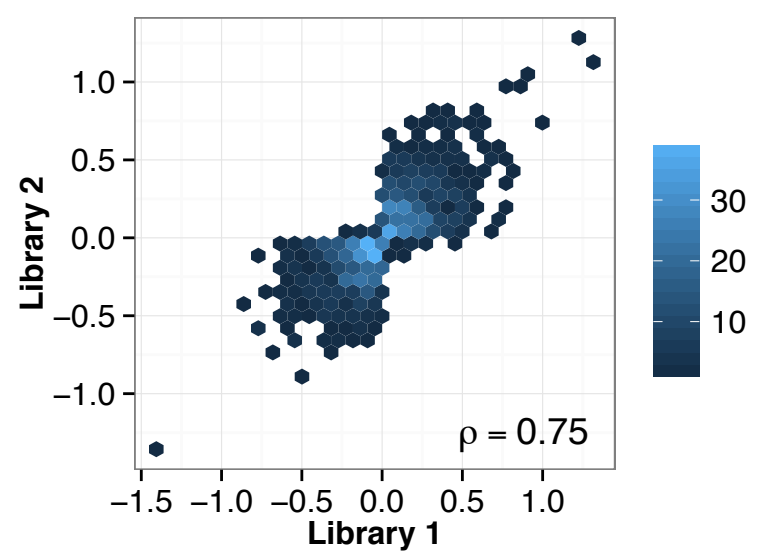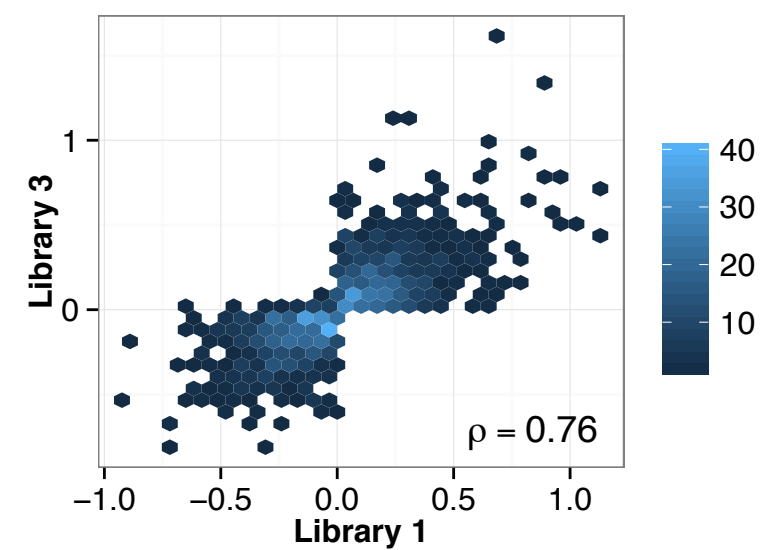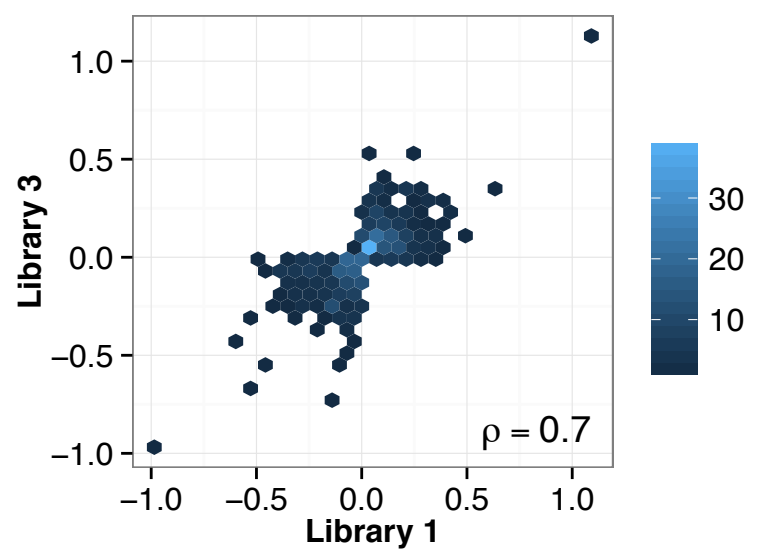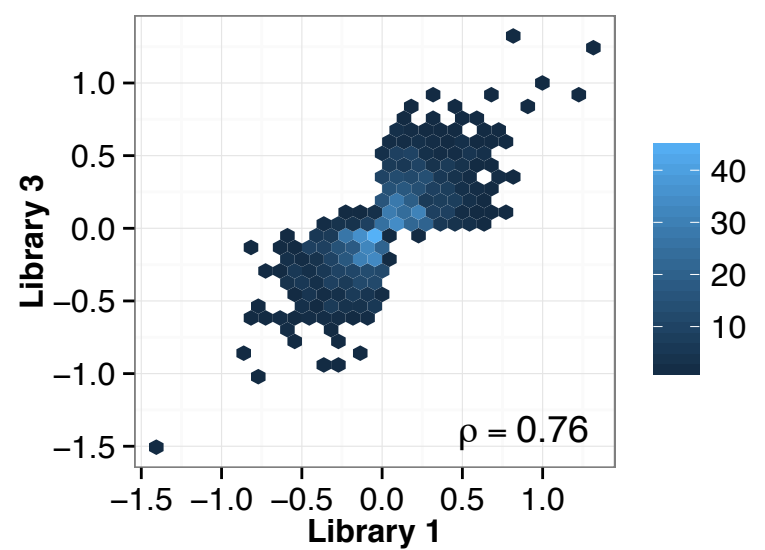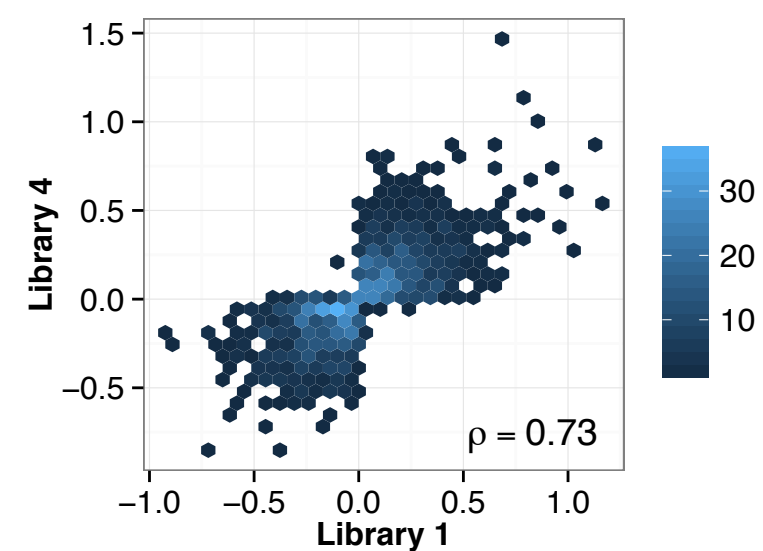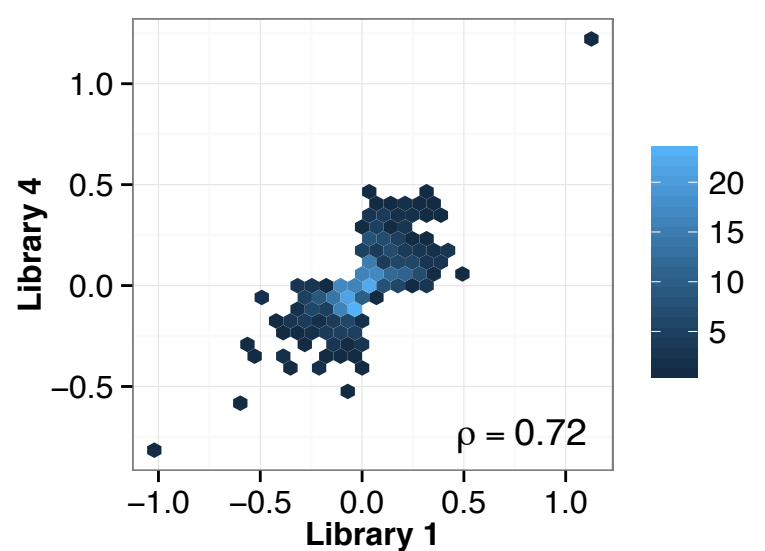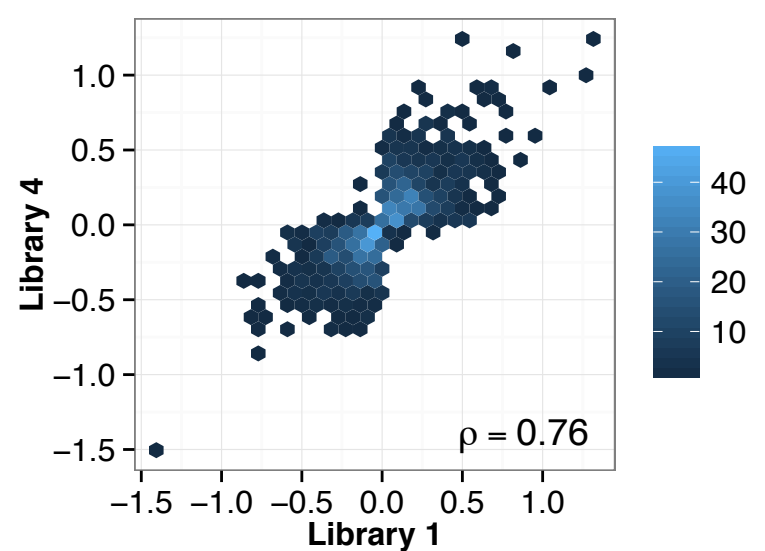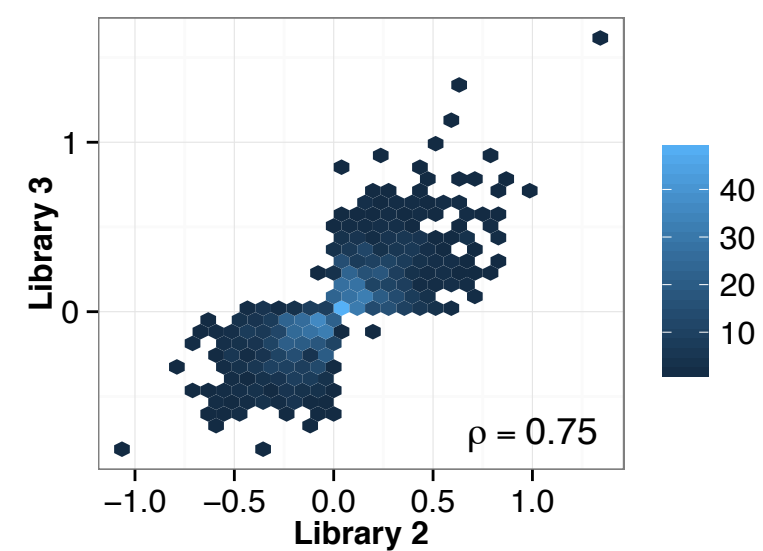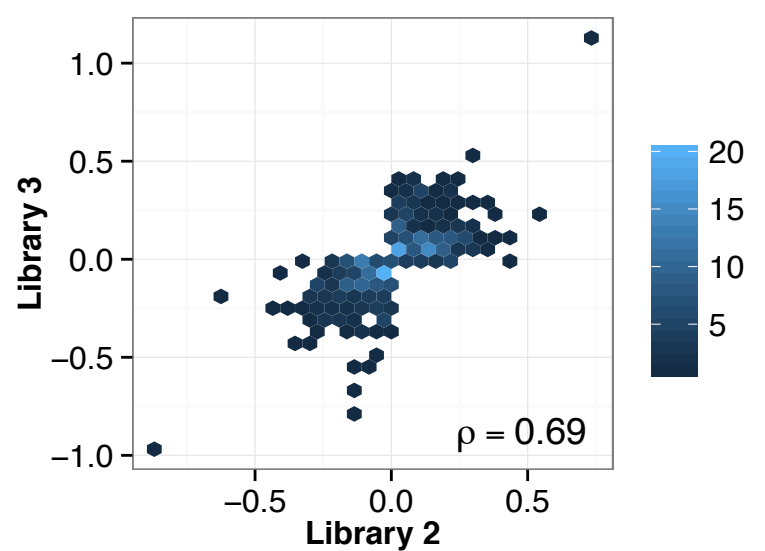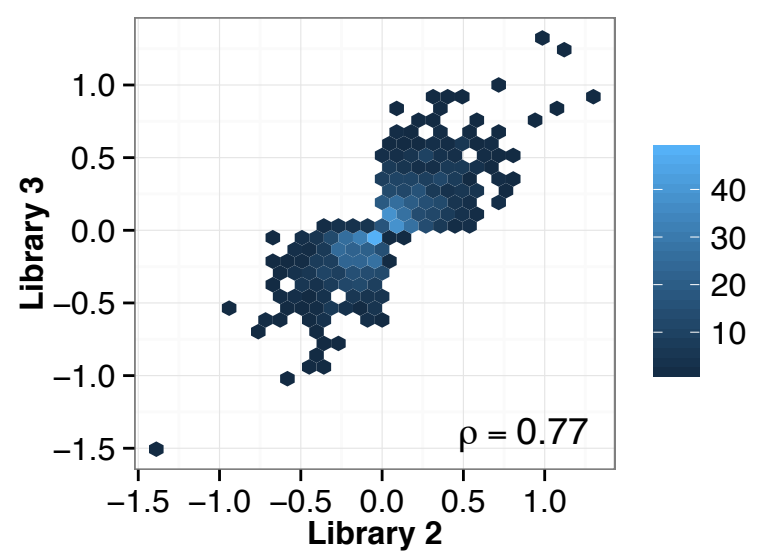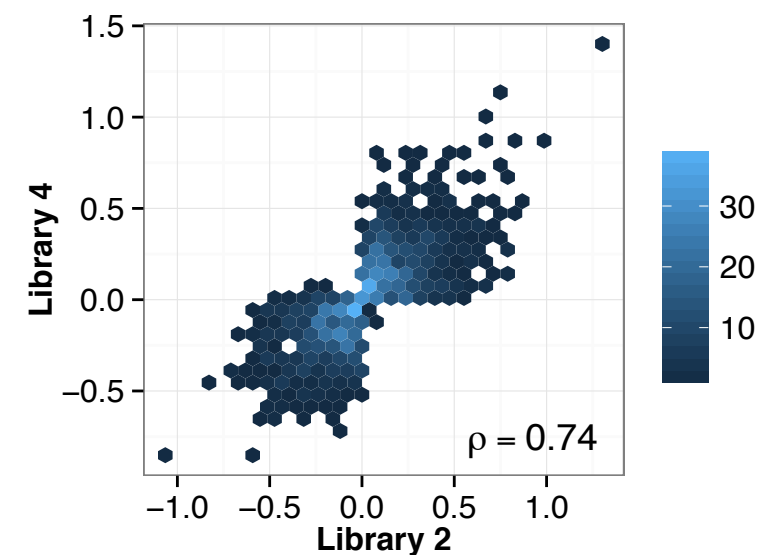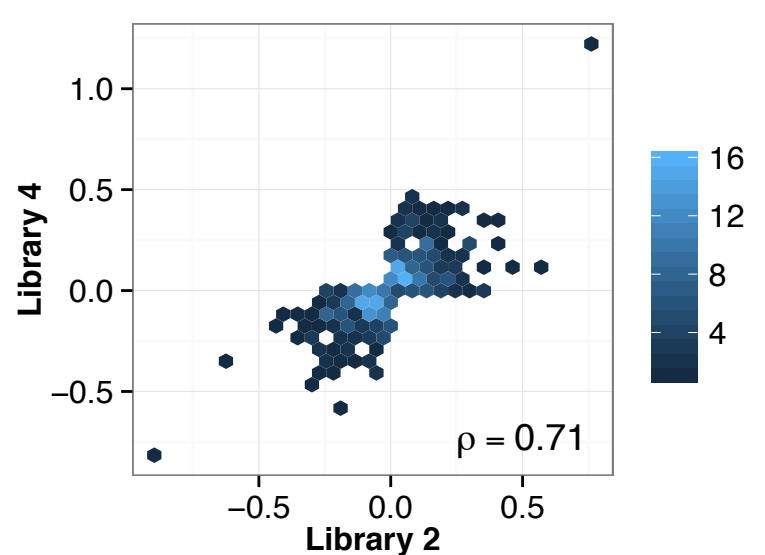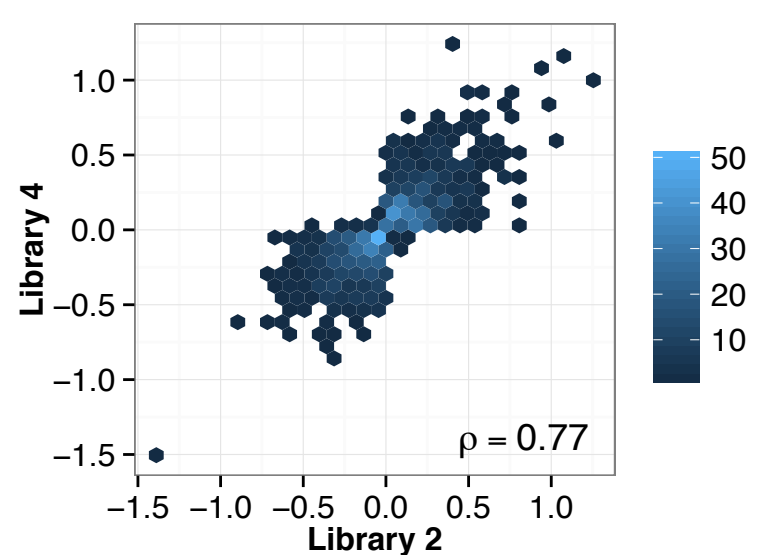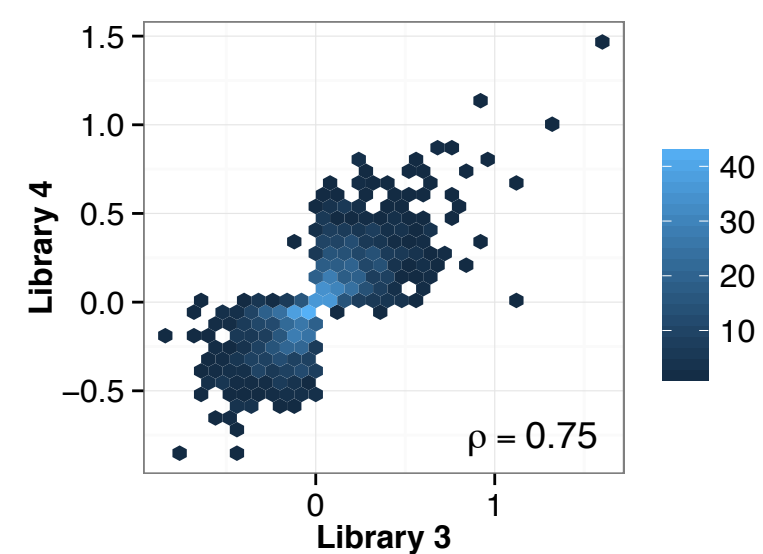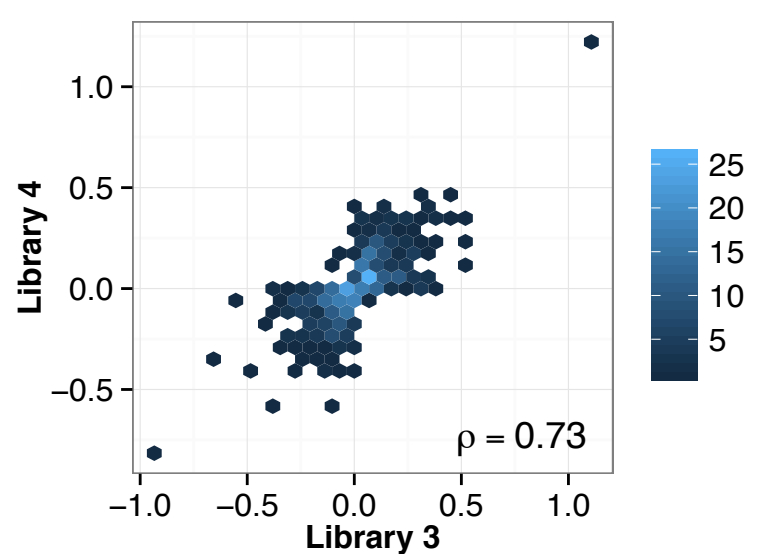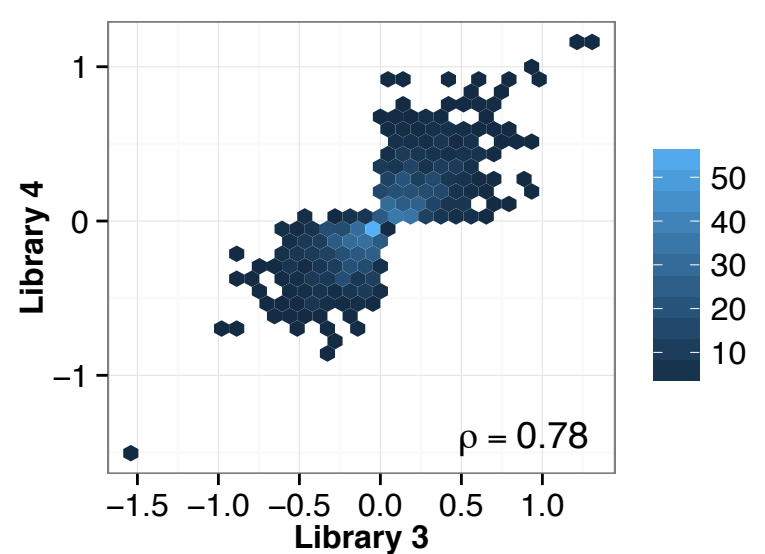

Supplement: Supplementary file 5 — Pairwise GSP comparisons reveal high concordance between four Qiagen sub-libraries. Lighter color indicates higher number of points per hexagon. (PDF 429 kb) [file 13059_2015_783_MOESM5_ESM.pdf]

## B. abortus

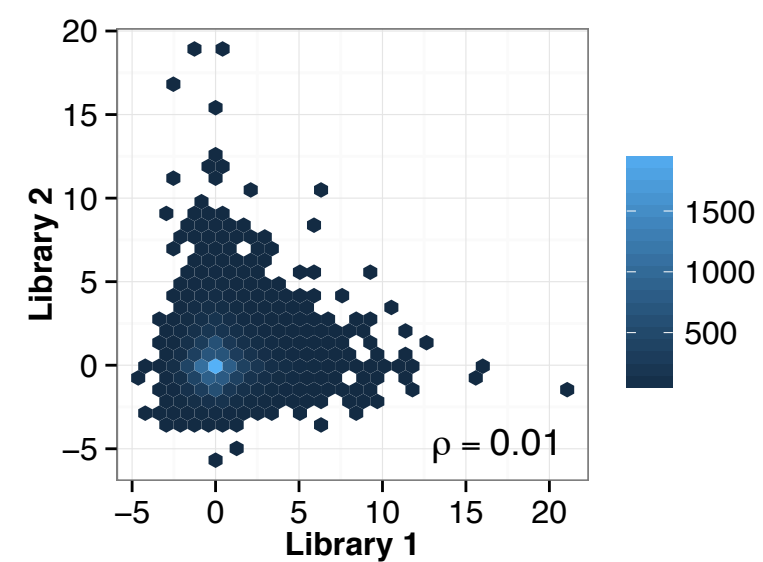

### B. henselae

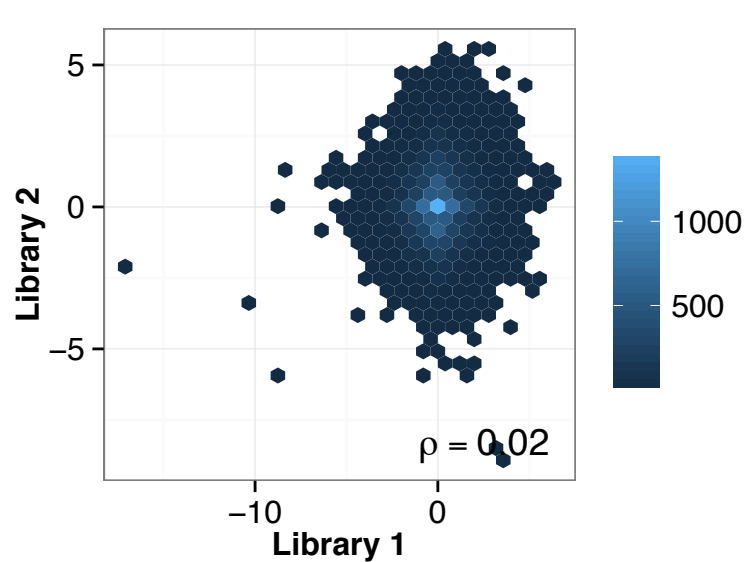

*S. typhimurium*

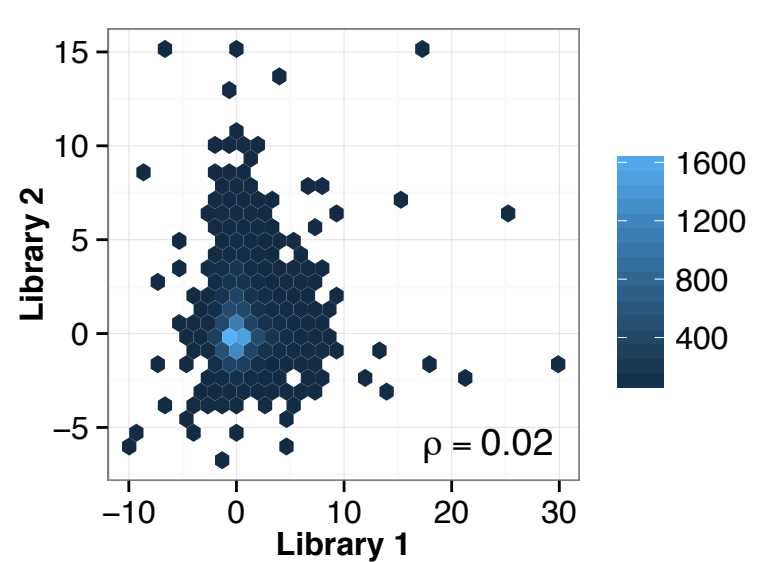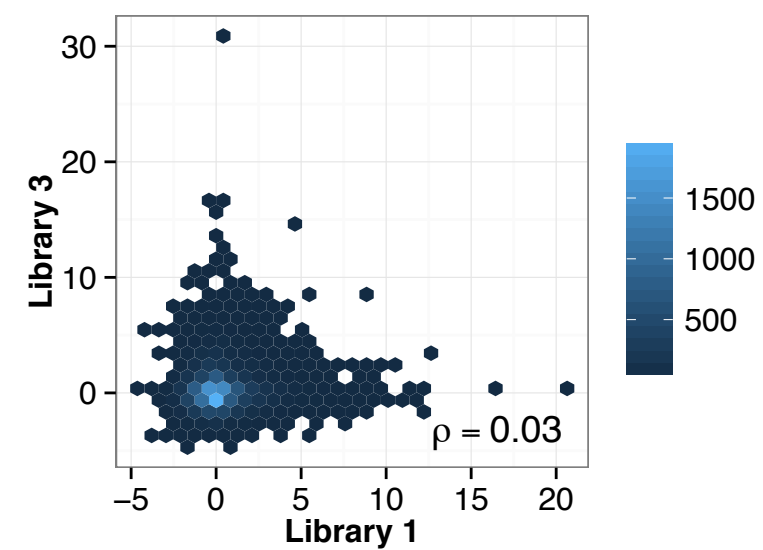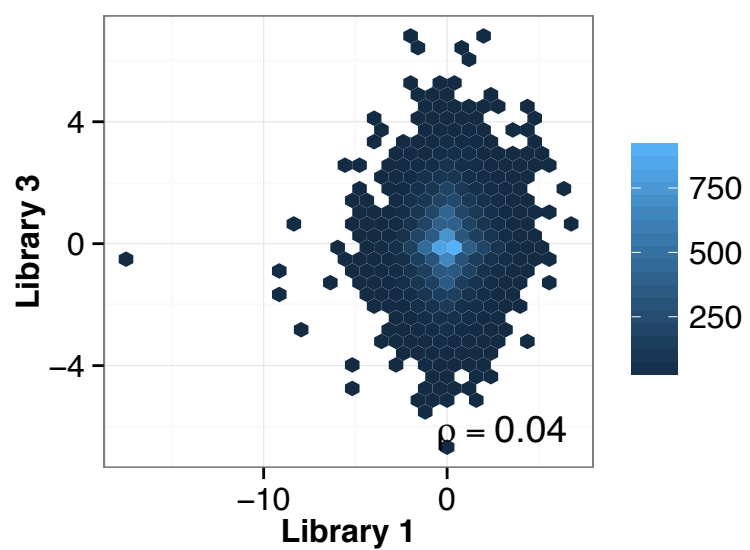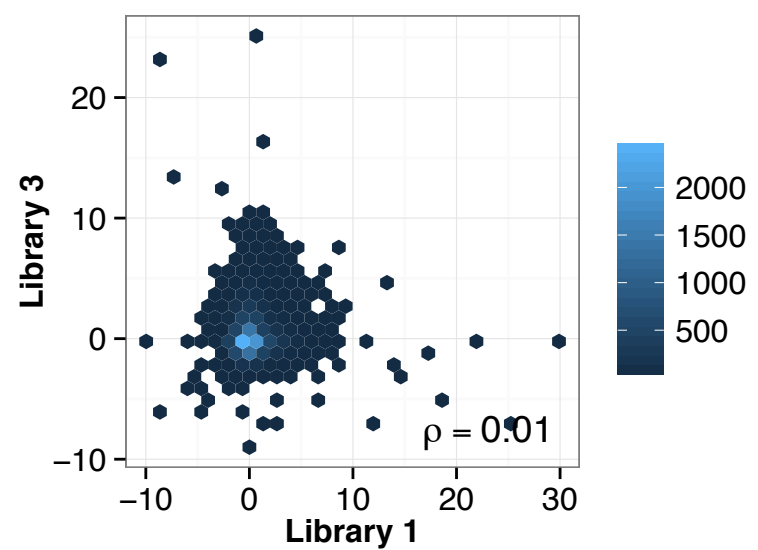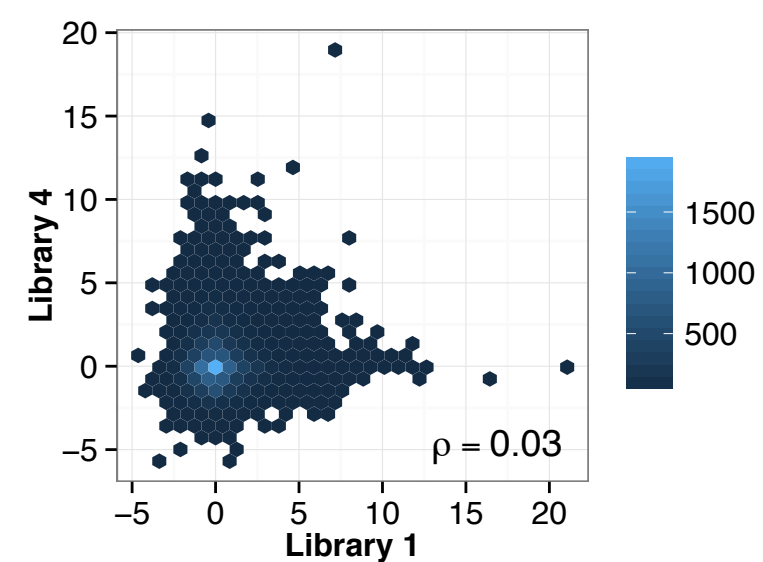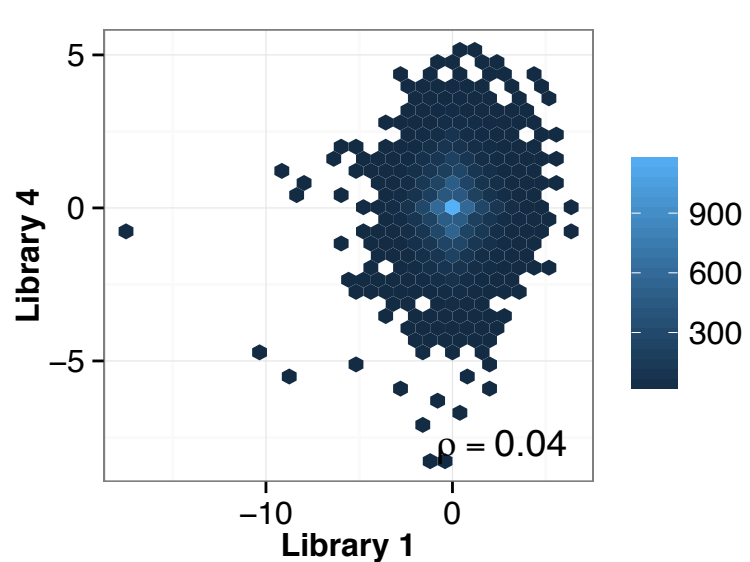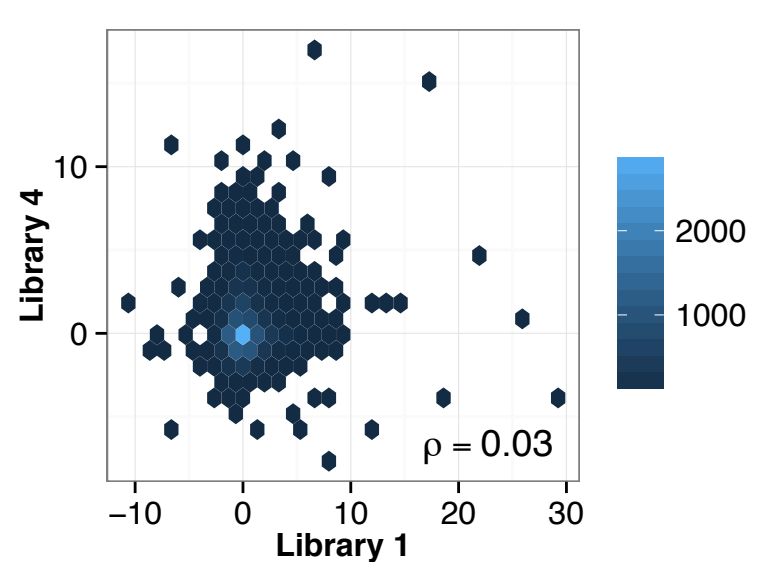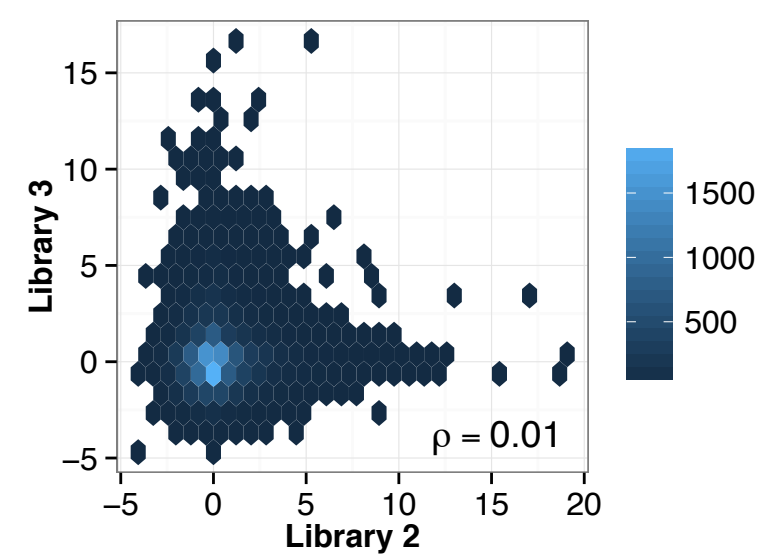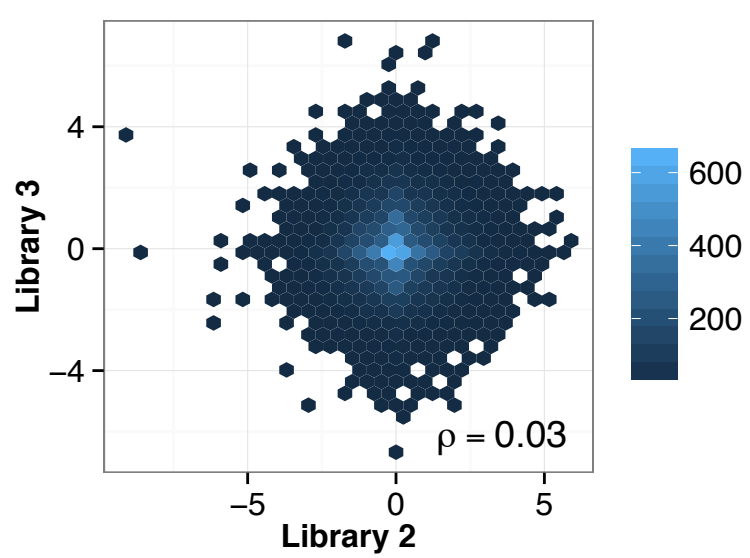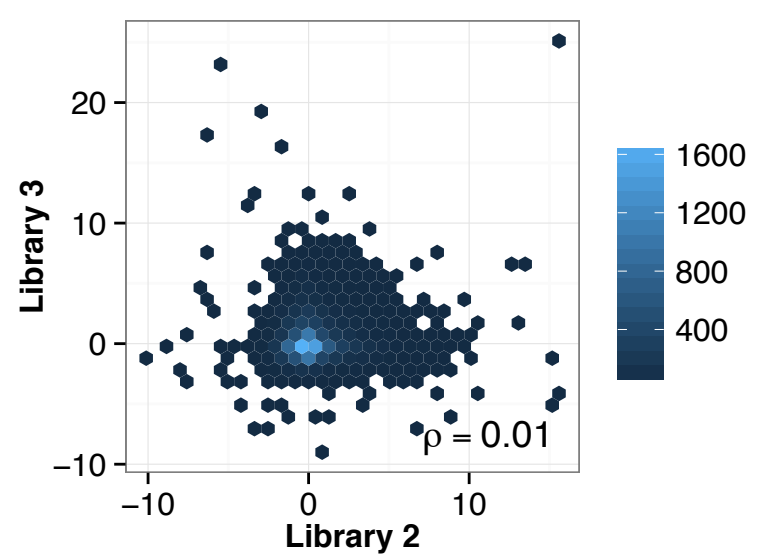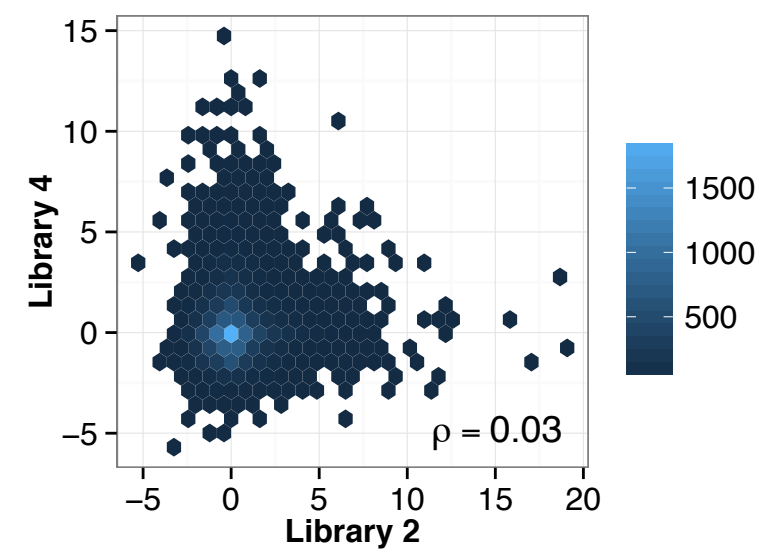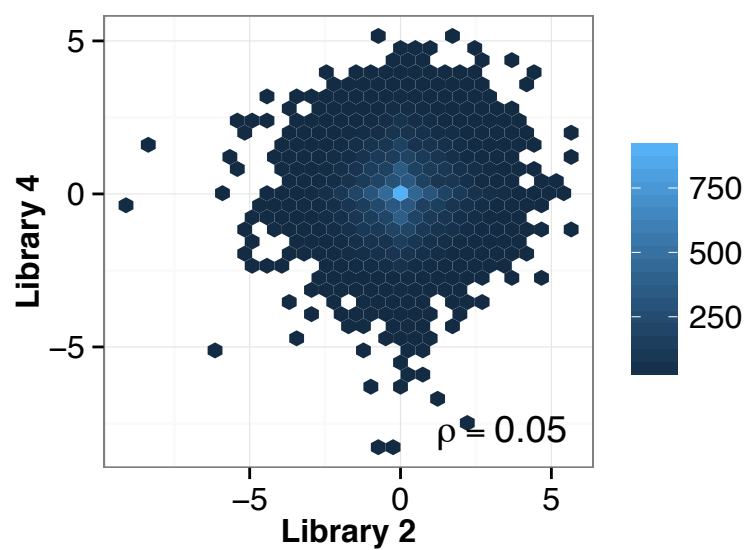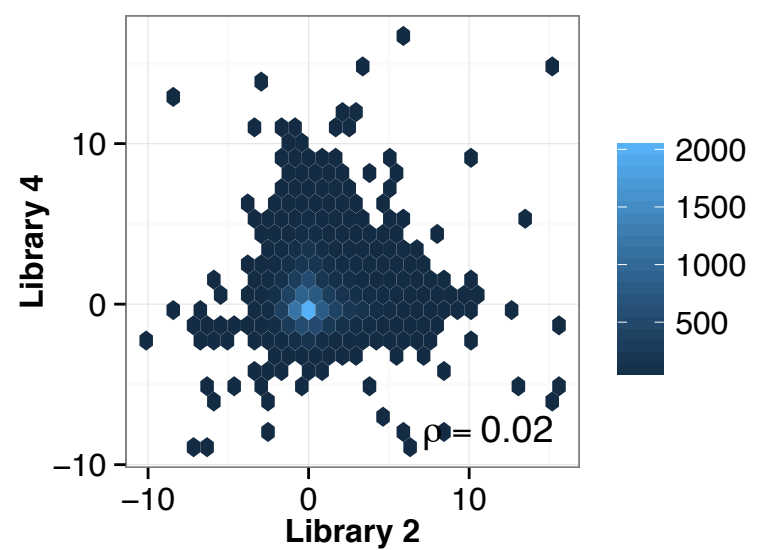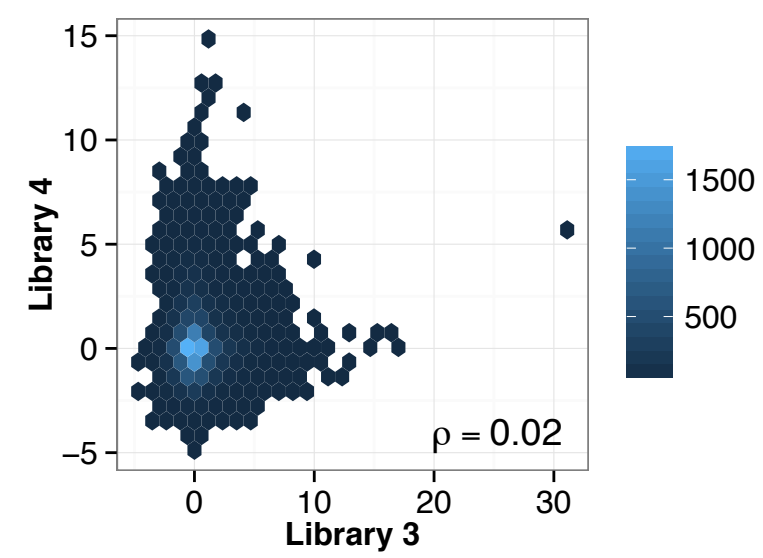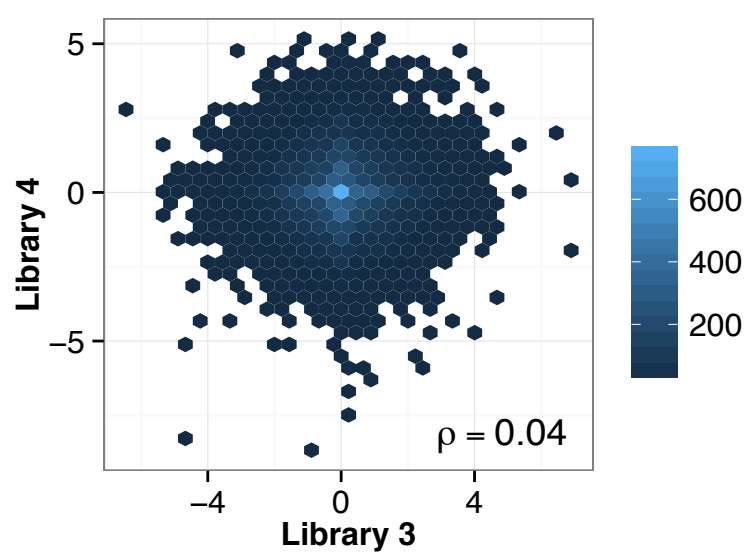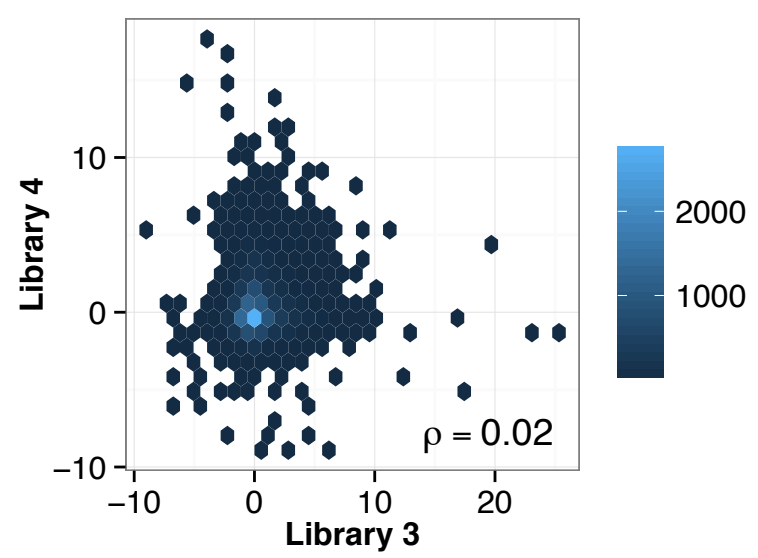

Supplement: Supplementary file 6 — Pairwise RSP comparisons reveal low concordance between four Qiagen sub-libraries. Lighter color indicates higher number of points per hexagon. (PDF 474 kb) [file 13059_2015_783_MOESM6_ESM.pdf]

**a**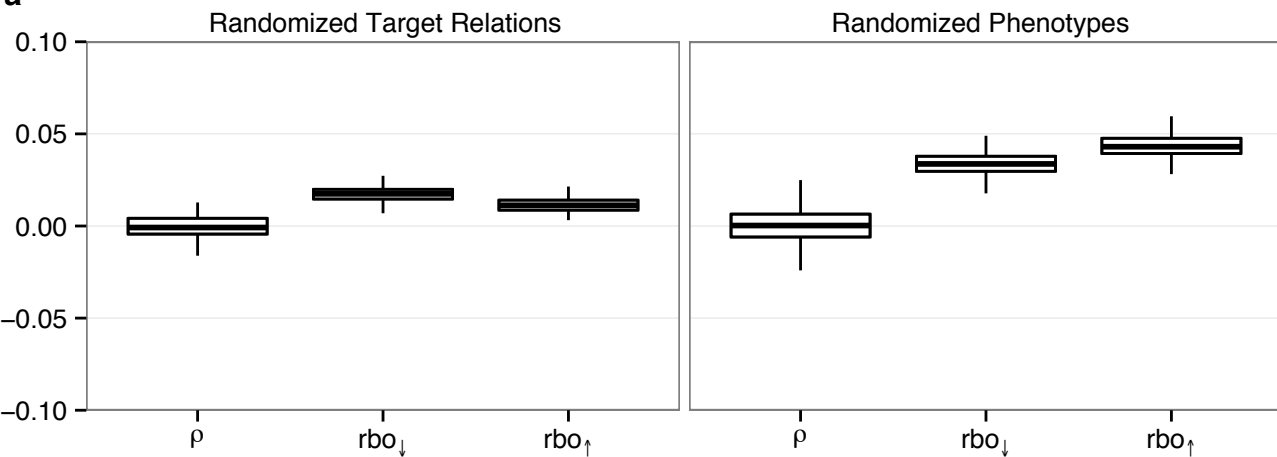**b**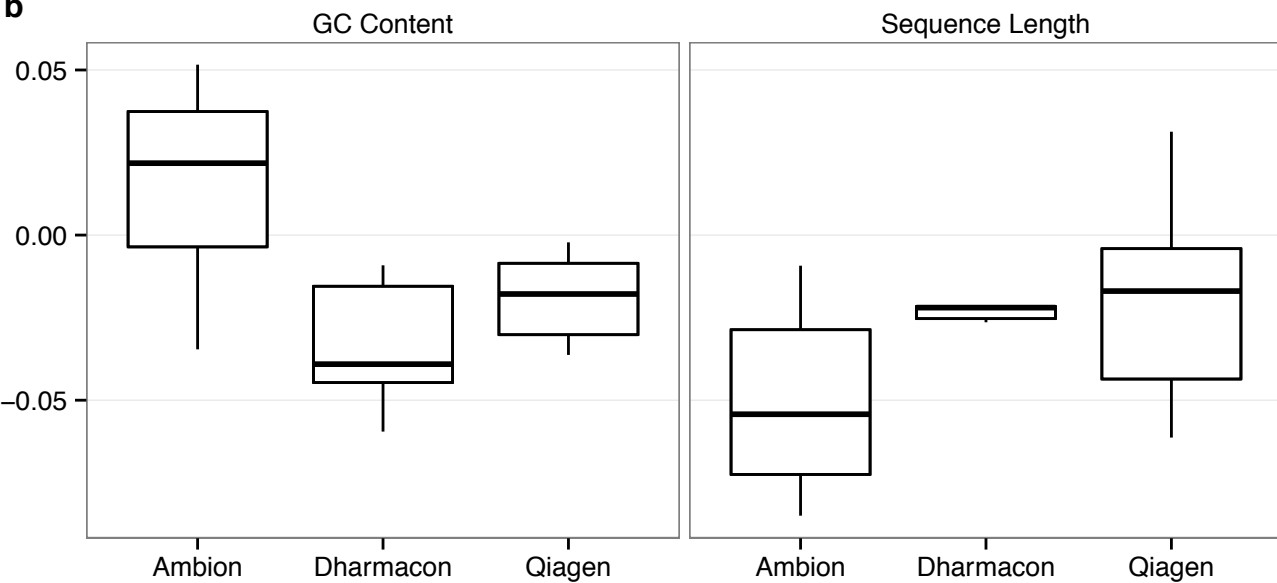

Supplement: Supplementary file 7 — gespeR GSPs are not concordant for randomized data and do not correlate with GC content or length of 3’ UTR transcripts. a Concordance between gene-specific phenotypes estimated from randomized siRNA-to-gene target relation matrices (covariate matrices; left), and from observed siRNA-specific phenotypes (response vectors; right). Correlation and rank-biased overlap were throughout close to zero, indicating that no spurious concordance is introduced when gespeR is fit. Results shown are from the B. abortus Qiagen screen, but were similar for other pathogens and libraries. b Correlation between estimated gene-specific phenotypes (GSPs) and GC content (left) and length of transcript 3' UTRs (right). Across libraries, estimated phenotype correlation was close to zero, indicating that GC content and the length of 3' UTRs do not confound the estimation of GSPs. (PDF 145 kb) [file 13059_2015_783_MOESM7_ESM.pdf]

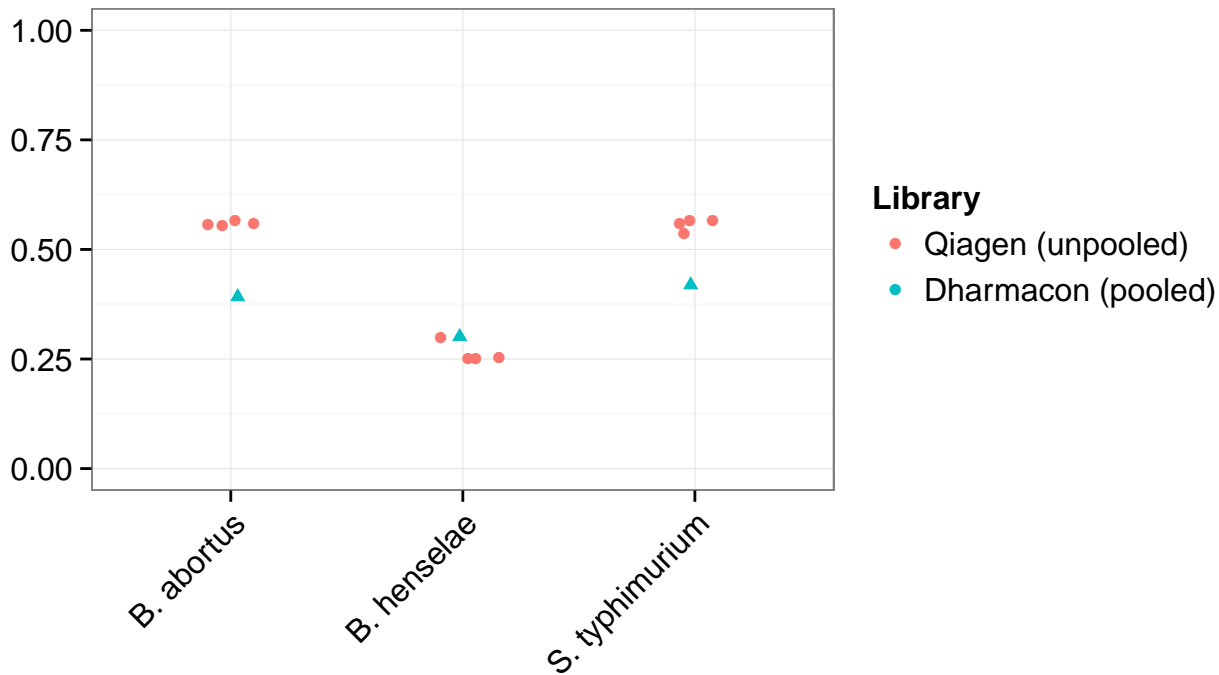

Supplement: Supplementary file 8 — Coefficients of determination (R2) for gespeR GSP estimates from data from Qiagen unpooled libraries and the Dharmacon pooled library indicate respectable model fits. (PDF 5 kb) [file 13059_2015_783_MOESM8_ESM.pdf]

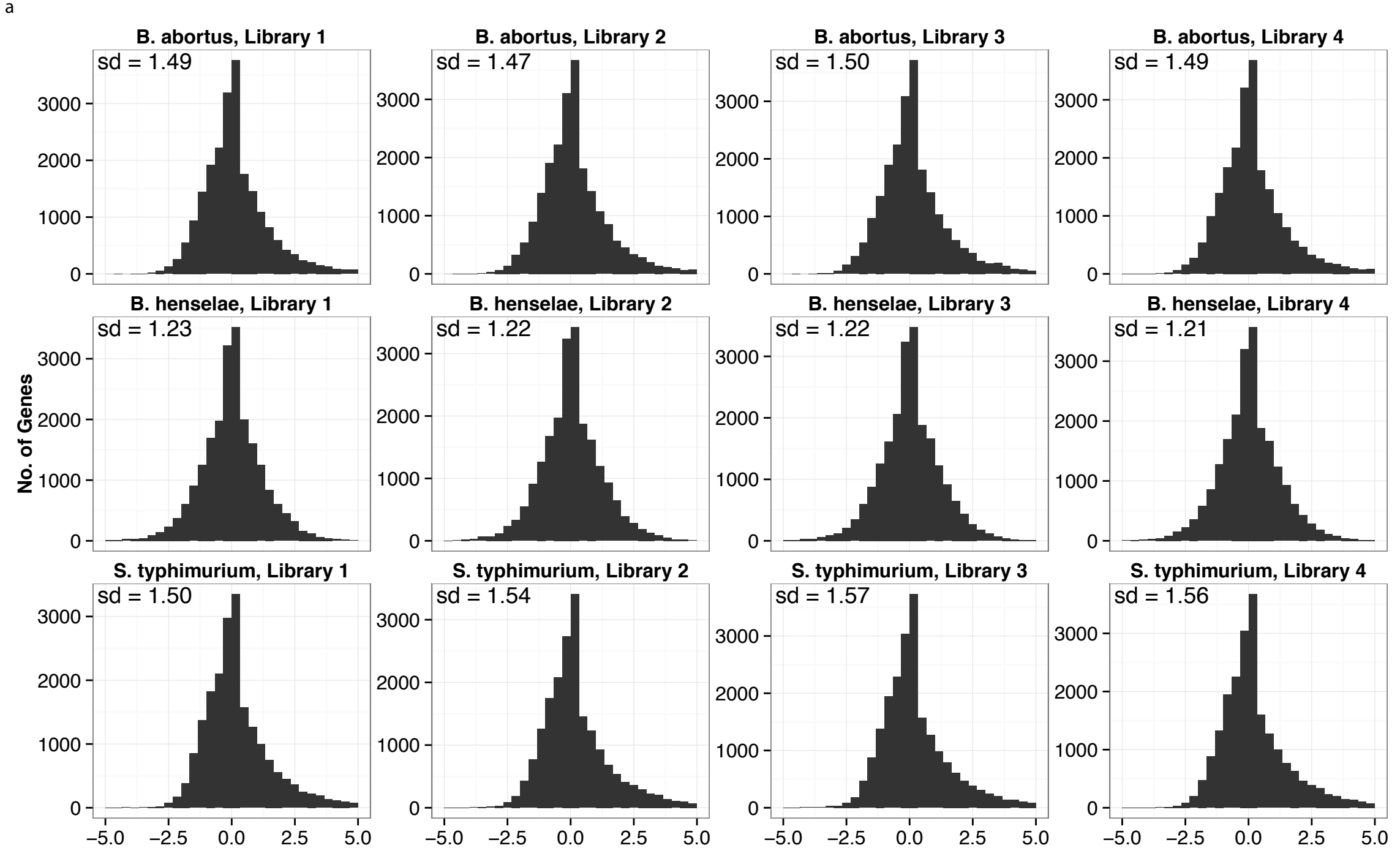

**b**

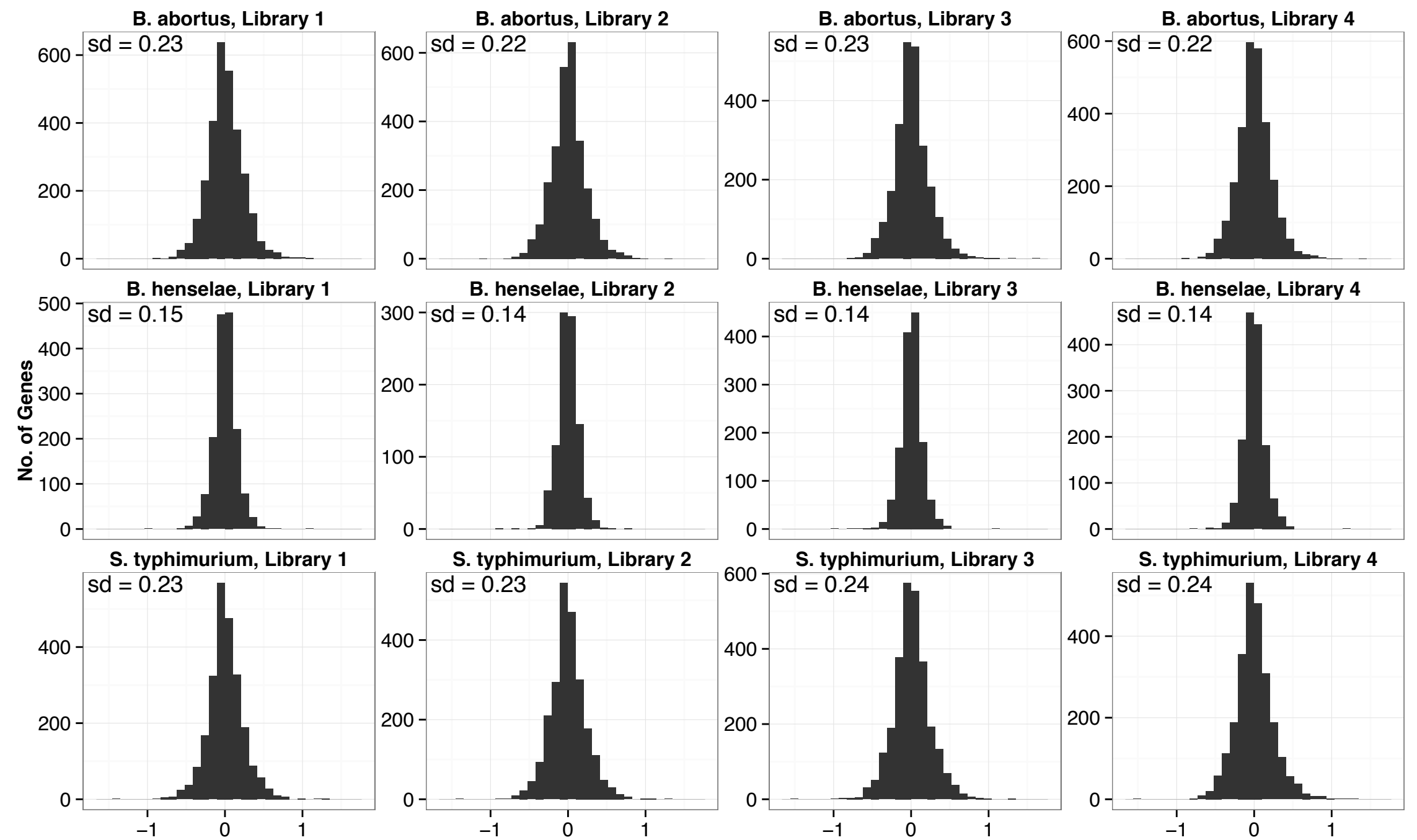

Supplement: Supplementary file 9 — Distributions for both GSPs and RSPs exhibit smaller variation for B. henselae compared with B. abortus and S. typhimurium. (PDF 251 kb) [file 13059_2015_783_MOESM9_ESM.pdf]

## False discovery rate

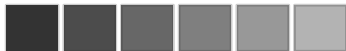

0.00 0.05 0.10 0.15 0.20 0.25

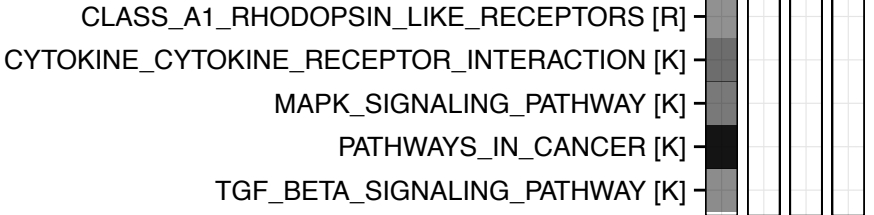

Supplement: Supplementary file 10 — Gene set enrichment analysis of GSP estimates for regulators of TGF-β signaling reveals five significantly enriched pathways with a false discovery rate (FDR) smaller than 0.25. Canonical pathway databases: R Reactome, K KEGG. GSPs estimated by gespeR are enriched for five pathways, including, as expected, TGF-β signaling. (PDF 127 kb) [file 13059_2015_783_MOESM10_ESM.pdf]

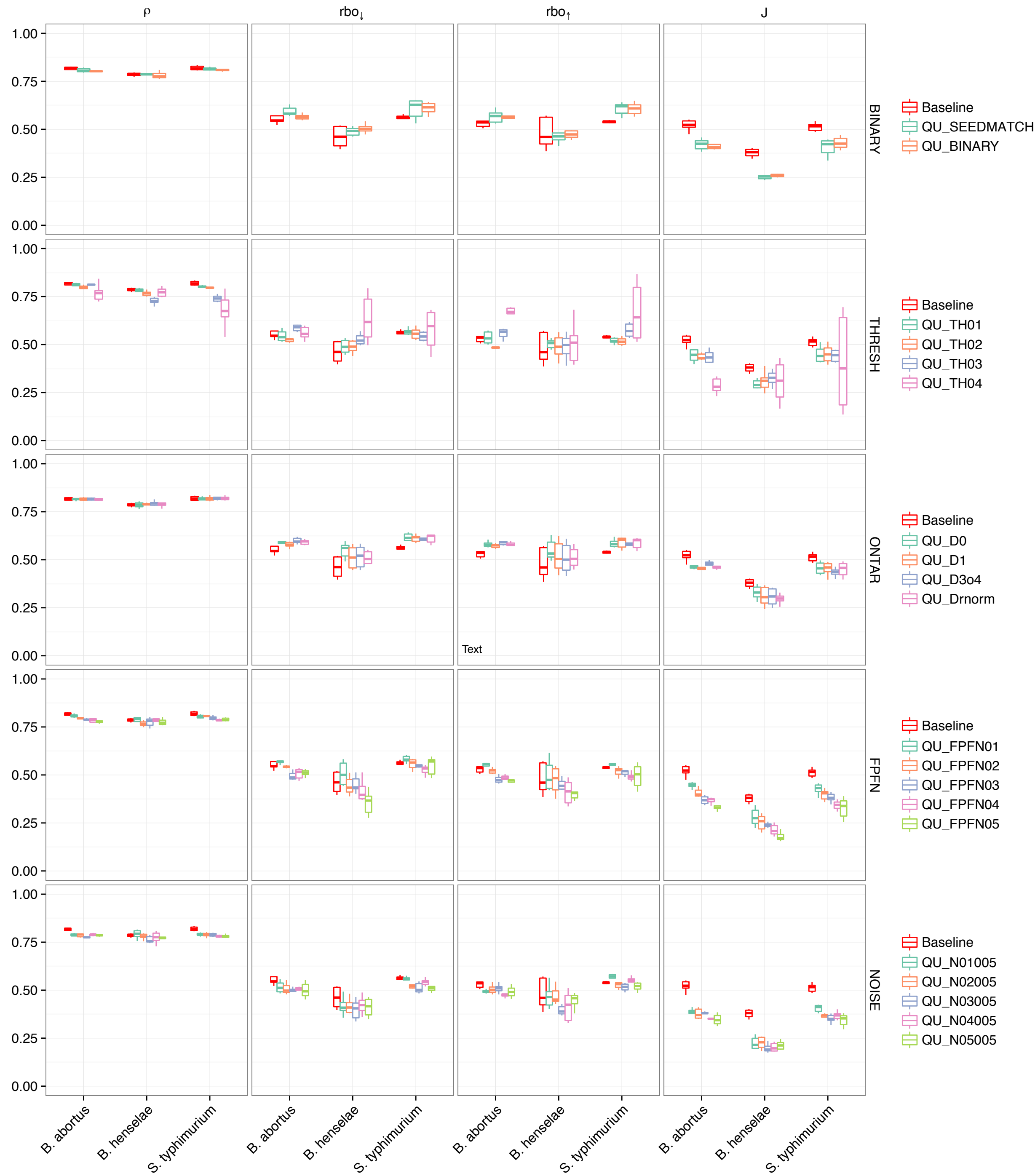

Supplement: Supplementary file 11 — Various alterations to gespeR’s reagent-to-target relation matrix reveal stability with respect to concordance between GSP estimates from different libraries. The tested variants of the reagent-to-target relation matrix, all of which disturb the original TargetScan predictions, include: QU_SEEDMATCH, binary seed-to-3’ UTR match indicator matrix which contains a 1 if the seed of an siRNA matches a 3’ UTR and 0 otherwise; QU_BINARY, binarized baseline Qiagen matrices; QU_TH0x, thresholded matrices, with only off-targets stronger than 0.x included; QU_D0, on-target component removed; QU_D1, on-target component set to 100 % knockdown efficacy; QU_D3o4, randomly kept three or four out of four on-target components at 75 % and set the remaining ones to 0; QU_Drnorm, sampled on-target components from N(0.75, 0.1); QU_FPFN0x, swapped 10× percentage of predicted targets with predicted non-targets; QU_N0x005, added N(0.x, 0.05) Gaussian noise added to all predicted targets. (PDF 65 kb) [file 13059_2015_783_MOESM11_ESM.pdf]

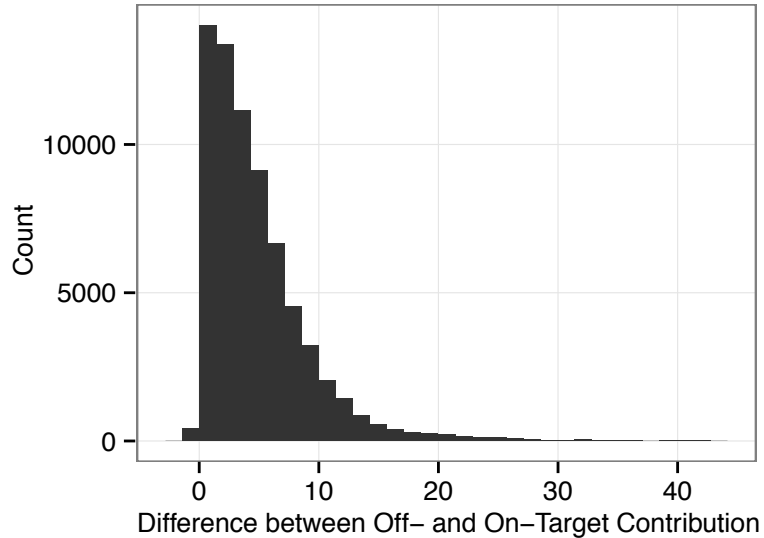

Supplement: Supplementary file 12 — Off-targeted genes dominate observed reagent-specific phenotypes. The gespeR model was fit to the B. abortus phenotypes from the Qiagen screen. On- and off-target contributions to the observed RSP for each reagent i = 1 … n were calculated as con,i = xij * β for the on-targeted gene j and coff,i = Yi – con,i – εi. The distribution of coff,i – con,i exhibits a strong positive tail, indicating that, in general, the combined contribution to the RSP from all off-targeted genes exceeds the contribution from the on-targeted gene. (PDF 98 kb) [file 13059_2015_783_MOESM12_ESM.pdf]

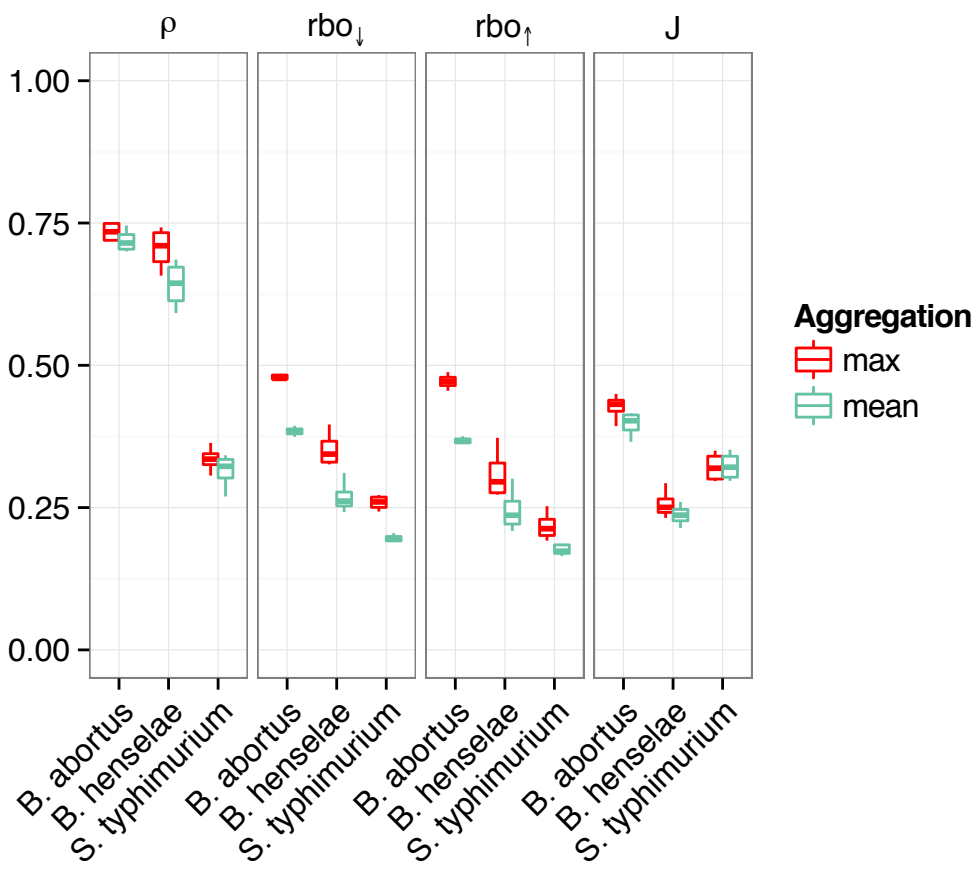

Supplement: Supplementary file 13 — Maximum aggregation of joint off-target effects for siRNA pools leads to increased concordance compared with arithmetic mean aggregation. (PDF 146 kb) [file 13059_2015_783_MOESM13_ESM.pdf]
