# Supplementary material for: gespeR: a statistical model for deconvoluting off-target-confounded RNA interference screens
Source: Genome Biol. 2015 Oct 7;16:220. doi: 10.1186/s13059-015-0783-1 (PMC4597449; doi:10.1186/s13059-015-0783-1)
Supplement: Supplementary file 3 — Prediction of RSPs using the gespeR model. Reagent-specific phenotypes Y(p) i are predicted by matrix multiplication of GSP estimates β(m) j from one data set (top) with reagent-specific target relations X(p) ij for another data set (bottom). (PDF 298 kb) [file 13059_2015_783_MOESM3_ESM.pdf]

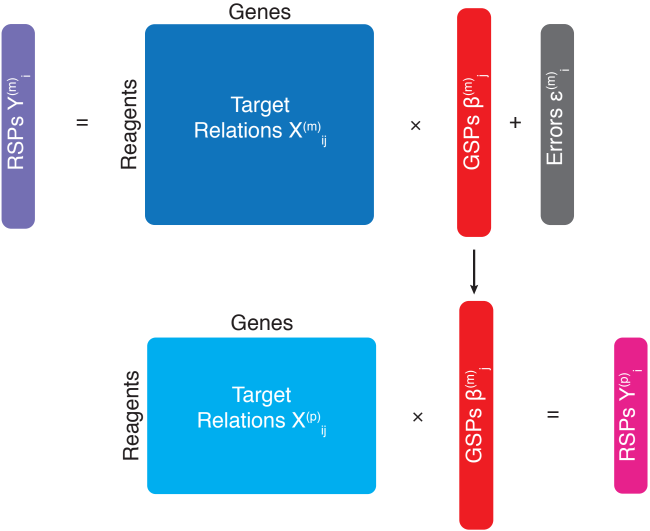
RSPs $Y^{(m)}_i$
=
Genes
Reagents
Target Relations $X^{(m)}_{ij}$
×
GSPs $\beta^{(m)}_j$
+
Errors $\varepsilon^{(m)}_i$
Genes
Reagents
Target Relations $X^{(p)}_{ij}$
×
GSPs $\beta^{(m)}_j$
=
RSPs $Y^{(p)}_i$
