# Supplementary material for: gespeR: a statistical model for deconvoluting off-target-confounded RNA interference screens
Source: Genome Biol. 2015 Oct 7;16:220. doi: 10.1186/s13059-015-0783-1 (PMC4597449; doi:10.1186/s13059-015-0783-1)
Supplement: Supplementary file 4 — Supplementary material [ 15 , 16 , 31 , 34 , 41 , 42 ]. Table S1 Top 50 hits for Brucella abortus ranked by absolute value of Infectivity GSPs. PubMed PMIDs are provided for previously reported components. Table S2 Top 50 hits for Bartonella henselae ranked by absolute value of Infectivity GSPs. PubMed PMIDs are provided for previously reported components. Table S3 Top 50 hits for Salmonella typhimurium ranked by absolute value of Infectivity GSPs. PubMed PMIDs are provided for previously reported components. Table S4 Top 50 hits for regulators of TGF-β signaling ranked by absolute value of GSPs. PubMed PMIDs are provided for previously reported components. gespeR identifies known components of the TGF-β pathway not identified in the original study due to confounding off-target effects. (DOCX 49 kb) [file 13059_2015_783_MOESM4_ESM.docx]

**Supplementary Material**

**Limited applicability of RSA and haystack**

RSA provides a ranking for gene prioritisation, but is not designed to predict unseen phenotypic readout. Moreover, RSA requires at least two readouts stemming from distinct reagents per gene or otherwise degenerates to simple ranking by individual knockdown phenotypes. Therefore, comparison to RSA is not possible for the evaluation of predictive performance and concordance between the Dharmacon pooled and Qiagen unpooled libraries.

Haystack’s target relation matrix is seed-based. Therefore, haystack cannot be applied to phenotypic data from pooled siRNA screens, where target predictions for the joint set of multiple siRNAs are required. A possible extension of haystack would be to aggregate seed-based target relation matrices in a similar fashion as it was done for the gespeR siRNA-to-gene target matrices (see section Reagent-to-gene target relations *x_ij_*)

**Application of gespeR to randomised data**

In order to test whether gespeR artificially introduces concordance between phenotypes, we fitted our model to randomised data. We obtained randomised phenotypic readout with the same distribution as the original data by random assignment of siRNA labels to phenotypes. siRNA-to-gene target relations (covariate matrices) were randomised keeping column sums and row sums fixed. Following our previous analysis for evaluating concordance, we found that both correlation and rbo were close to zero, indicating that no spurious concordance is introduced using the gespeR model.

**gespeR’s performance under alterations to the siRNA-to-gene target relation**

We performed numerous *in silico* experiments to evaluate the effects of alterations to the siRNA-to-gene target relation matrix on the performance of our model. The following alterations, disturbing the original prediction of on- and off-targeting strengths, were implemented based on the four genome-wide Qiagen sub-libraries. Subsequently, changes in performances compared to the baseline without alterations were evaluated (**Additional file 11: Figure S10**).

**Binarisation**

siRNA-to-gene target relation matrices were binarised, where each value larger than a threshold of 0, 0.1, 0.2, 0.3, and 0.4 was set to 1, and otherwise to 0. This led to increasingly sparse matrices, which contained only predicted off-targets above certain strength. In addition, we constructed a simple binary seed match matrix, where a target relation is set to 1 when the 7mer seed matches the 3’ UTR of a gene, and 0 otherwise. We found that binarisation of the matrices had only a minor effect on the performance of gespeR. The most notable difference is a drop in performance with respect to the overall overlap between GSP estimates (Jaccard index). The rank-biased overlap (rbo) was slightly increased compared to the baseline.

**Thresholding**

We also investigated, whether target predictions with a weak effect on transcript abundance contribute to the performance of our model and thresholded the matrices, removing increasingly more target relations. In general, this led to decreased performance, except for the rbo measures, where very strong thresholds seem to result in higher concordance. This observation is explained by the fact that only very few genes are in fact selected at this level (< 10), which leads strong variation. Increasing the trehshold, such that only predicted targets with a strong effect of more than 50% transcript abundance are included did not allow to fit the model anymore.

**Disturbing the on-target**

In order to investigate the effect of the siRNA on-target, we altered the on-target component of the matrices. We (a) removed the on-target component completely by setting it to 0 for all siRNAs, (b) set the on-target component to 1, corresponding to the maximum strength of 100% transcript knockdown for all siRNAs (instead of 75%), (c) randomly removed one or two of the total four on-target components for one gene, simulating the case where only three or four out of four siRNAs have functioning on-targets and (d) sampled the on-target component from N(0.75, 0.1), simulating differential on-target performance of individual siRNAs. The alterations to the on-target component do not have a very strong impact on the performance of our model, in line with our previous observations of dominating off-target effects.

**Addition of false positives and false negatives**

We evaluated gespeR’s performance after the addition of false positive and false negatives to the siRNA-to-gene target relation matrices. In this experiment, we randomly selected an increasingly larger subset of targets for each siRNA (cutoffs = 0.1, 0.2, 0.3, 0.4 and 0.5) and swapped it with a random selection of non-targets. In this way, we simultaneously increased the number of false negative and false positive target predictions. We observed a clear decrease in performance when we increased the number of false positives and false negatives.

**Addition of Gaussian noise**

A similar effect to adding false positives and false negatives was observed, when we added increasing amounts of Gaussian noise (mean = 0.1, 0.2, 0.3, 0.4, 0.5; sd = 0.05) to the strength of the predicted target relations.

**Software**

The results presented in this article were obtained using R version 3.1.1 and software packages glmnet 1.9.8, dplyr 0.2, reshape2 1.4, ggplot2 1.0.0, doMC 1.3.3 and biomaRt 2.20.0. siRNA target prediction was performed using TargetScan 6.2 [31, 34]. TargetScan output was parsed and transformed using Python 2.7.5. Haystack [16] and RSA [15] implementations were downloaded in March 2013 and April 2014, respectively from the authors’ websites [41, 42] and applied with default parameters (lower and upper bound thresholds for RSA were set to -1 and +1, respectively).

**Supplementary Figures**

**Supplementary Figure 1**. Distribution of the number of off-targets per siRNA for different strength cut-offs.

**Supplementary Figure 2**. Removal of row and column effects using B score normalisation [18] illustrated for a 384 well plate from the B. abortus screen.

**Supplementary Figure 3**. Prediction of RSPs using the gespeR model.

**Supplementary Figure 4**. Pairwise GSP comparisons reveal high concordance between four Qiagen sub-libraries.

**Supplementary Figure 5**. Pairwise RSP comparisons reveal low concordance between four Qiagen sub-libraries.

**Supplementary Figure 6**. gespeR GSPs are not concordant for randomised data and do not correlate with GC content or length of 3’ UTR transcripts.

**Supplementary Figure 7**. Coefficients of determination (R2) for gespeR GSP estimates from data from Qiagen unpooled libraries and the Dharmacon pooled library indicate respectable model fits.

**Supplementary Figure 8**. Distributions for both GSPs and RSPs exhibit smaller variation for B. henselae compared to B. abortus and S. typhimurium.

**Supplementary Figure 9**. Gene Set Enrichment Analysis of GSP estimates for regulators of TGF-β signaling reveals five significantly enriched pathways.

**Supplementary Figure 10**. Variations to gespeR’s target relation matrix reveals stability of concordance between GSP estimates from different libraries.

**Supplementary Figure 11**. Off-targeted genes dominate observed reagent-specific phenotypes.

**Supplementary Figure 12**. Maximum aggregation of joint off-target effects for siRNA pools leads to increased concordance compared to arithmetic mean aggregation.

**Supplementary Tables**

Top-50 hits for *Brucella abortus*

| Rank | ID | Symbol | GSP | Function | Reference [PMID] |
| --- | --- | --- | --- | --- | --- |
| 1 | 6625 | SNRNP70 | 1.579 |  |  |
| 2 | 11157 | LSM6 | 1.434 |  |  |
| 3 | 51593 | SRRT | 1.315 |  |  |
| 4 | 64784 | CRTC3 | 1.280 |  |  |
| 5 | 4781 | NFIB | 1.212 |  |  |
| 6 | 195828 | ZNF367 | -1.179 |  |  |
| 7 | 441502 | RPS26P11 | 1.064 |  |  |
| 8 | 7756 | ZNF207 | 1.034 |  |  |
| 9 | 375 | ARF1 | -1.032 | Golgi to ER transport | 19557163 |
| 10 | 51571 | FAM49B | 1.029 |  |  |
| 11 | 3758 | KCNJ1 | 1.027 |  |  |
| 12 | 10787 | NCKAP1 | -1.025 | Actin dynamics | 11672528, 11579087 |
| 13 | 2948 | GSTM4 | -1.014 |  |  |
| 14 | 85014 | TMEM141 | 1.004 |  |  |
| 15 | 372 | ARCN1 | -0.970 | Golgi to ER transport | 19557163 |
| 16 | 136319 | MTPN | 0.962 | Actin dynamics | 12488317, 11579087 |
| 17 | 388276 | C16orf97 | 0.947 |  |  |
| 18 | 10657 | KHDRBS1 | 0.929 |  |  |
| 19 | 998 | CDC42 | -0.927 | Actin dynamics | 11579087 |
| 20 | 284307 | ZIK1 | -0.914 |  |  |
| 21 | 84193 | SETD3 | -0.911 |  |  |
| 22 | 10160 | FARP1 | 0.901 | Actin dynamics | 2320930, 11579087 |
| 23 | 391194 | OR2M2 | 0.895 |  |  |
| 24 | 7046 | TGFBR1 | -0.891 |  |  |
| 25 | 284611 | FAM102B | 0.888 |  |  |
| 26 | 6118 | RPA2 | 0.887 |  |  |
| 27 | 5587 | PRKD1 | 0.886 |  |  |
| 28 | 3792 | KEL | 0.880 |  |  |
| 29 | 6181 | RPLP2 | 0.877 |  |  |
| 30 | 103910 | MYL12B | 0.877 |  |  |
| 31 | 29 | ABR | 0.874 |  |  |
| 32 | 6633 | SNRPD2 | 0.871 |  |  |
| 33 | 56947 | MFF | 0.867 |  |  |
| 34 | 6231 | RPS26 | 0.859 |  |  |
| 35 | 6631 | SNRPC | 0.856 |  |  |
| 36 | 1047 | CLGN | 0.854 |  |  |
| 37 | 5908 | RAP1B | 0.845 |  |  |
| 38 | 1998 | ELF2 | -0.843 |  |  |
| 39 | 6428 | SRSF3 | 0.827 |  |  |
| 40 | 284615 | ANKRD34A | 0.816 |  |  |
| 41 | 100534611 | NA | -0.810 |  |  |
| 42 | 55731 | FAM222B | 0.801 |  |  |
| 43 | 6416 | MAP2K4 | -0.800 |  |  |
| 44 | 10458 | BAIAP2 | 0.798 | Actin dynamics | 11130076, 11579087 |
| 45 | 9424 | KCNK6 | 0.792 |  |  |
| 46 | 100506033 | PTOV1-AS1 | 0.784 |  |  |
| 47 | 9848 | MFAP3L | 0.781 |  |  |
| 48 | 146956 | EME1 | 0.771 |  |  |
| 49 | 9069 | CLDN12 | 0.766 |  |  |
| 50 | 89797 | NAV2 | -0.763 |  |  |

**Supplementary Table 1**. Top-50 hits for *Brucella abortus* ranked by absolute value of *Infectivity* GSPs. PubMed (PMIDs) are provided for previously reported components.

Top-50 hits for *Bartonella henselae*

| Rank | ID | Symbol | GSP | Function | Reference [PMID] |
| --- | --- | --- | --- | --- | --- |
| 1 | 11157 | LSM6 | 1.460 |  |  |
| 2 | 3678 | ITGA5 | -1.249 | Integrin signaling: Binding partner of ITGB1 | 19693543 |
| 3 | 2033 | EP300 | -1.100 |  |  |
| 4 | 6935 | ZEB1 | -0.902 |  |  |
| 5 | 7027 | TFDP1 | 0.770 |  |  |
| 6 | 3688 | ITGB1 | -0.740 | Invasome formation | 22045736 |
| 7 | 7127 | TNFAIP2 | -0.676 |  |  |
| 8 | 6231 | RPS26 | 0.674 |  |  |
| 9 | 114294 | LACTB | -0.619 |  |  |
| 10 | 4627 | MYH9 | 0.606 | Actin-associated motor protein | 23295347 |
| 11 | 10736 | SIX2 | 0.599 |  |  |
| 12 | 79856 | SNX22 | 0.564 |  |  |
| 13 | 6667 | SP1 | 0.543 |  |  |
| 14 | 8061 | FOSL1 | -0.538 |  |  |
| 15 | 258 | AMBN | -0.538 |  |  |
| 16 | 10927 | SPIN1 | 0.529 |  |  |
| 17 | 55819 | RNF130 | -0.520 |  |  |
| 18 | 55294 | FBXW7 | -0.518 |  |  |
| 19 | 1642 | DDB1 | 0.512 |  |  |
| 20 | 6134 | RPL10 | 0.508 |  |  |
| 21 | 201780 | SLC10A4 | 0.506 |  |  |
| 22 | 7094 | TLN1 | -0.497 |  |  |
| 23 | 51115 | RMDN1 | 0.495 |  |  |
| 24 | 282706 | DAOA-AS1 | 0.494 |  |  |
| 25 | 441502 | RPS26P11 | 0.493 |  |  |
| 26 | 11148 | HHLA2 | 0.492 |  |  |
| 27 | 55752 | SEPT11 | 0.482 |  |  |
| 28 | 5885 | RAD21 | 0.480 |  |  |
| 29 | 113730 | KLHDC7B | -0.477 |  |  |
| 30 | 2314 | FLII | -0.476 |  |  |
| 31 | 90957 | DHX57 | -0.467 |  |  |
| 32 | 29968 | PSAT1 | -0.460 |  |  |
| 33 | 6421 | SFPQ | -0.459 |  |  |
| 34 | 5937 | RBMS1 | -0.459 |  |  |
| 35 | 463 | ZFHX3 | -0.455 |  |  |
| 36 | 80135 | RPF1 | 0.451 |  |  |
| 37 | 150384 | GTSE1-AS1 | -0.438 |  |  |
| 38 | 23383 | MAU2 | 0.437 |  |  |
| 39 | 6628 | SNRPB | 0.435 |  |  |
| 40 | 87 | ACTN1 | -0.434 |  |  |
| 41 | 1539 | CYLC2 | 0.434 |  |  |
| 42 | 90025 | UBE3D | -0.433 |  |  |
| 43 | 79633 | FAT4 | 0.425 |  |  |
| 44 | 6633 | SNRPD2 | 0.424 |  |  |
| 45 | 57819 | LSM2 | 0.421 |  |  |
| 46 | 51564 | HDAC7 | -0.419 |  |  |
| 47 | 7416 | VDAC1P1 | 0.418 |  |  |
| 48 | 27125 | AFF4 | -0.415 |  |  |
| 49 | 9454 | HOMER3 | -0.407 |  |  |
| 50 | 3204 | HOXA7 | -0.407 |  |  |

**Supplementary Table 2**. Top-50 hits for *Bartonella henselae* ranked by absolute value of *Infectivity* GSPs. PubMed (PMIDs) are provided for previously reported components.

Top-50 hits for *Salmonella typhimurium*

| Rank | ID | Symbol | GSP | Function | Reference |
| --- | --- | --- | --- | --- | --- |
| 1 | 4659 | PPP1R12A | 1.696 |  |  |
| 2 | 998 | CDC42 | -1.444 | Actin dynamics | 8953049, 9630225 |
| 3 | 7871 | SLMAP | 1.350 |  |  |
| 4 | 374739 | TEPP | 1.202 |  |  |
| 5 | 283651 | HMGN2P46 | 1.185 |  |  |
| 6 | 90835 | C16orf93 | 1.130 |  |  |
| 7 | 158763 | ARHGAP36 | 1.101 |  |  |
| 8 | 1072 | CFL1 | 1.037 | Actin dynamics | 15056216, 14992720 |
| 9 | 372 | ARCN1 | -1.031 | Golgi to ER transport | 21407211 |
| 10 | 5949 | RBP3 | 1.001 |  |  |
| 11 | 3685 | ITGAV | 0.999 |  |  |
| 12 | 5660 | PSAP | -0.986 |  |  |
| 13 | 5732 | PTGER2 | 0.952 |  |  |
| 14 | 27092 | CACNG4 | -0.951 |  |  |
| 15 | 4147 | MATN2 | 0.910 |  |  |
| 16 | 2064 | ERBB2 | 0.896 |  |  |
| 17 | 64784 | CRTC3 | -0.891 |  |  |
| 18 | 51324 | SPG21 | 0.889 |  |  |
| 19 | 6059 | ABCE1 | 0.876 |  |  |
| 20 | 4627 | MYH9 | -0.873 | Actin dynamics | 21501827 |
| 21 | 407835 |  | 0.863 |  |  |
| 22 | 832 | CAPZB | 0.853 |  |  |
| 23 | 401089 | FOXL2NB | 0.851 |  |  |
| 24 | 153396 | TMEM161B | 0.844 |  |  |
| 25 | 8826 | IQGAP1 | 0.844 | Rac1 & CDC42 signaling | 17693642 |
| 26 | 170063 | CXorf22 | -0.833 |  |  |
| 27 | 400713 | ZNF880 | 0.829 |  |  |
| 28 | 64778 | FNDC3B | 0.827 |  |  |
| 29 | 125893 | ZNF816 | 0.826 |  |  |
| 30 | 1849 | DUSP7 | 0.818 |  |  |
| 31 | 29953 | TRHDE | -0.816 |  |  |
| 32 | 9788 | MTSS1 | 0.813 |  |  |
| 33 | 169792 | GLIS3 | -0.813 |  |  |
| 34 | 3837 | KPNB1 | 0.811 |  |  |
| 35 | 23607 | CD2AP | 0.809 |  |  |
| 36 | 55410 |  | 0.804 |  |  |
| 37 | 1984 | EIF5A | -0.795 |  |  |
| 38 | 394263 | MUC21 | -0.793 |  |  |
| 39 | 131890 | GRK7 | 0.791 |  |  |
| 40 | 144717 | FAM109A | 0.788 |  |  |
| 41 | 25973 | PARS2 | 0.776 |  |  |
| 42 | 306 | ANXA3 | 0.774 |  |  |
| 43 | 51134 | CEP83 | -0.773 |  |  |
| 44 | 100216001 | LINC00704 | 0.762 |  |  |
| 45 | 54946 | SLC41A3 | 0.757 |  |  |
| 46 | 79686 | NA | -0.755 |  |  |
| 47 | 132789 | GNPDA2 | 0.752 |  |  |
| 48 | 7764 | ZNF217 | -0.743 |  |  |
| 49 | 10216 | PRG4 | -0.742 |  |  |
| 50 | 158434 |  | 0.736 |  |  |

**Supplementary Table 3**. Top-50 hits for *Salmonella typhimurium* ranked by absolute value of *Infectivity* GSPs. PubMed (PMIDs) are provided for previously reported components.

Top-50 hits for TGF-β signaling

| *Rank* | *ID* | *Symbol* | *GSP* | *Function* | *Reference* |
| --- | --- | --- | --- | --- | --- |
| 1 | 7048 | TGFBR2 | -0.736 | Upstream modulator | 21401928 |
| 2 | 56990 | CDC42SE2 | -0.123 |  |  |
| 3 | 100288805 | HYDIN2 | 0.122 |  |  |
| 4 | 92482 | BBIP1 | -0.115 |  |  |
| 5 | 9534 | ZNF254 | -0.114 |  |  |
| 6 | 3751 | KCND2 | 0.112 |  |  |
| 7 | 57154 | SMURF1 | 0.110 | Regulator of SMAD | 11278251 |
| 8 | 7046 | TGFBR1 | -0.107 | Upstream modulator | 21401928 |
| 9 | 89857 | KLHL6 | -0.105 |  |  |
| 10 | 85416 | ZIC5 | -0.104 |  |  |
| 11 | 8492 | PRSS12 | 0.104 |  |  |
| 12 | 9732 | DOCK4 | 0.102 |  |  |
| 13 | 11148 | HHLA2 | 0.100 |  |  |
| 14 | 7849 | PAX8 | -0.099 |  |  |
| 15 | 158747 | MOSPD2 | -0.098 |  |  |
| 16 | 220074 | LRTOMT | -0.097 |  |  |
| 17 | 1964 | EIF1AX | 0.096 |  |  |
| 18 | 135112 | NCOA7 | -0.095 |  |  |
| 19 | 7132 | TNFRSF1A | -0.094 |  |  |
| 20 | 285 | ANGPT2 | 0.093 |  |  |
| 21 | 8292 | COLQ | 0.093 |  |  |
| 22 | 100379174 | MACROD2-AS1 | -0.091 |  |  |
| 23 | 1618 | DAZL | 0.091 |  |  |
| 24 | 80746 | TSEN2 | -0.089 |  |  |
| 25 | 23467 | NPTXR | 0.086 |  |  |
| 26 | 54602 | NDFIP2 | 0.085 |  |  |
| 27 | 163223 | ZNF676 | 0.085 |  |  |
| 28 | 6364 | CCL20 | -0.084 |  |  |
| 29 | 989 | SEPT7 | -0.083 |  |  |
| 30 | 100533464 | PRH1-PRR4 | 0.083 |  |  |
| 31 | 29993 | PACSIN1 | -0.083 |  |  |
| 32 | 57654 | UVSSA | 0.082 |  |  |
| 33 | 11183 | MAP4K5 | -0.082 |  |  |
| 34 | 79109 | MAPKAP1 | 0.082 |  |  |
| 35 | 6658 | SOX3 | -0.082 |  |  |
| 36 | 220296 | HEPACAM | -0.081 |  |  |
| 37 | 56890 | MDM1 | -0.081 |  |  |
| 38 | 55709 | KBTBD4 | -0.080 |  |  |
| 39 | 387723 | LINC00959 | 0.080 |  |  |
| 40 | 390980 | ZNF805 | -0.080 |  |  |
| 41 | 221400 | TDRD6 | 0.080 |  |  |
| 42 | 403314 | APOBEC4 | -0.079 |  |  |
| 43 | 134429 | STARD4 | 0.078 |  |  |
| 44 | 80731 | THSD7B | 0.078 |  |  |
| 45 | 23598 | PATZ1 | 0.077 |  |  |
| 46 | 8864 | PER2 | 0.077 |  |  |
| 47 | 285889 | AC073133.1 | -0.077 |  |  |
| 48 | 81790 | RNF170 | -0.076 |  |  |
| 49 | 56681 | SAR1A | -0.076 |  |  |
| 50 | 374393 | FAM111B | -0.076 |  |  |

**Supplementary Table 4**. Top-50 hits for regulators of TGF-β signaling ranked by absolute value of GSPs. PubMed (PMIDs) are provided for previously reported components. gespeR identifies known components of the TGF-β pathway not identified in the original study due to confounding off-target effects.
